# Transcriptome Analysis of the Signalling Networks in Coronatine-Induced Secondary Laticifer Differentiation from Vascular Cambia in Rubber Trees

**DOI:** 10.1038/srep36384

**Published:** 2016-11-03

**Authors:** Shaohua Wu, Shixin Zhang, Jinquan Chao, Xiaomin Deng, Yueyi Chen, Minjing Shi, Wei-Min Tian

**Affiliations:** 1 Ministry of Agriculture Key Laboratory of Biology and Genetic Resources of Rubber Tree/State Key Laboratory Breeding Base of Cultivation and Physiology for Tropical Crops, Rubber Research Institute, Chinese Academy of Tropical Agricultural Sciences, Danzhou, Hainan 571737, P.R. China

## Abstract

The secondary laticifer in rubber tree (*Hevea brasiliensis* Muell. Arg.) is a specific tissue within the secondary phloem. This tissue differentiates from the vascular cambia, and its function is natural rubber biosynthesis and storage. Given that jasmonates play a pivotal role in secondary laticifer differentiation, we established an experimental system with jasmonate (JA) mimic coronatine (COR) for studying the secondary laticifer differentiation: in this system, differentiation occurs within five days of the treatment of epicormic shoots with COR. In the present study, the experimental system was used to perform transcriptome sequencing and gene expression analysis. A total of 67,873 unigenes were assembled, and 50,548 unigenes were mapped at least in one public database. Of these being annotated unigenes, 15,780 unigenes were differentially expressed early after COR treatment, and 19,824 unigenes were differentially expressed late after COR treatment. At the early stage, 8,646 unigenes were up-regulated, while 7,134 unigenes were down-regulated. At the late stage, the numbers of up- and down-regulated unigenes were 7,711 and 12,113, respectively. The annotation data and gene expression analysis of the differentially expressed unigenes suggest that JA-mediated signalling, Ca^2+^ signal transduction and the CLAVATA-MAPK-WOX signalling pathway may be involved in regulating secondary laticifer differentiation in rubber trees.

Cell differentiation continues to occur throughout the lifetime of a plant. The development of different organs mainly depends on the stem cell balance within the root, shoot and axillary meristems of the plant. In perennial woody plants, vascular cambia, a type of secondary meristem, differentiate and produce the secondary phloem (which is located outside the vascular cambia, towards the exterior of the plant) and the secondary xylem, or wood, (which is located inside the vascular cambia)^1^,^2^. In recent years, great progress has been made in investigating both the secondary phloem as a whole and xylem development^3^,^4^,^5^, whereas little is known about the differentiation of specific tissues within the secondary phloem, owing to a lack of suitable markers for tracing specific tissue formation. Notably, the secondary metabolites that accumulate in the specific tissues within the secondary phloem are generally of economic importance, such as the resin in the resin ducts of Norway spruce (*Picea abies* L. Karst.)^6^ and the natural rubber in the secondary laticifers in the rubber tree (*Hevea brasiliensis* Muell. Arg.)^7^.

The secondary laticifer in rubber trees is a distinct tissue that is differentiated from the vascular cambia and is located in the secondary phloem. The number of secondary laticifers in the trunk bark of rubber trees is positively correlated with the natural rubber yield^7^. This number is clone-specific and is also influenced by environmental factors such as mechanical wounding and latex drainage^8^,^9^,^10^. An important finding was that jasmonates play a pivotal role in the differentiation of secondary laticifers^7^,^11^. The active form of jasmonate is (+)-7-iso-jasmonoyl-L-isoleucine (JA-Ile), not jasmonic acid or methyl jasmonate as it was long thought to be^12^. Given that coronatine (COR) can structurally and functionally mimic the active form of jasmonate^12^,^13^,^14^,^15^, we previously tested the effects of COR on the differentiation of secondary laticifers. We found that COR was much more effective than methyl jasmonate at inducing the differentiation of secondary laticifers^16^. The induced secondary laticifers were detected under a light microscope within five days of the epicormic shoots of rubber trees being treated with 20 μM COR^16^. Using this experimental system, suppression subtractive hybridization (SSH) suggested that genes involved in Ca^2+^ signal transduction participate in secondary laticifer differentiation^16^. Because plant cell differentiation is a complex process and may be regulated by multiple signals^17^, the data from the SSH library are limited and insufficient for revealing the regulatory networks in the secondary laticifer differentiation process. To gain further insight into the signalling networks involved in secondary laticifer differentiation from the vascular cambia in rubber trees, a global analysis of the inner bark transcriptome in response to COR was conducted using this experimental system.

## Results

### Effect of COR on secondary laticifer differentiation

**Figure 1:**
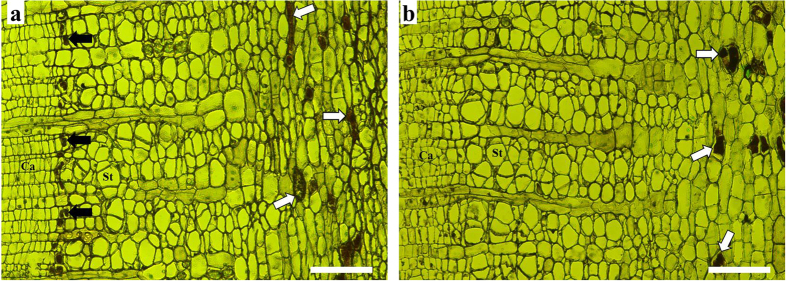
Light micrographs of bark cross-sections, showing secondary laticifer differentiation after COR treatment. (**a**) Treated with 20 μM COR. (**b**) Treated with water. White arrows show the primary laticifers. Black arrows show the secondary laticifers. Ca, cambia; St, sieve tube. Bars = 100 μm.

A row of secondary laticifers was visible in the secondary phloem of the epicormic shoots within five days of being treated with 20 μM COR (Fig. 1a). By contrast, water application (control) did not induce secondary laticifer differentiation (Fig. 1b). Moreover, the cell division in the region of vascular cambia was observed three days after JA treatment^7^. Therefore, genes that are differentially expressed within three days following COR treatment should be closely associated with the differentiation of secondary laticifers from the vascular cambia.

### Illumina sequencing and de novo assembly

The cDNA libraries from the Control-A, COR-A, Control-B and COR-B samples were separately sequenced on an Illumina HiSeq^TM^ 2000 platform and yielded a total of 347,550,238 raw RNA-seq reads. After removing low quality reads, approximately 80 million clean reads were obtained from each cDNA library (Table 1). With the aid of SOAPdenovo software, we obtained 130,199, 121,094, 143,435 and 131,686 contigs from Control-A, COR-A, Control-B and COR-B, respectively. The contigs were assembled into unigenes by paired-end assembly and gap-filling. This produced 75,468, 58,479, 71,994 and 58,907 unigenes from Control-A, COR-A, Control-B and COR-B, respectively. The combined analysis of these unigenes generated a total of 67,873 unigenes, referred to as “all unigenes”, with an average length of 750 nucleotides (nt) and an N50 of 1222 nt (Table 2). Of the 67,873 unigenes, 13,455 unigenes ranged in size from 1,000 to 2,000 nt and 5,359 unigenes were longer than 2,000 nt (Supplementary Fig. S1). The all unigenes set was used as reference transcriptome to annotate and analyze the differentially expressed genes (DEGs) between the COR- and water-treated samples.

### Functional annotation and classification of the transcriptome

Annotation and classification of unigenes is very important for understanding the transcriptome. In this study, the reference transcriptome (67,873 uingenes) were aligned with sequences in public databases, including the NCBI non-redundant protein database (NR), the Swiss-Prot protein database (Swiss-Prot, http://www.expasy.ch/sprot), the Kyoto Encyclopedia of Genes and Genomes (KEGG, http://www.genome.jp/kegg/), the Cluster of Orthologous Groups database (COG, http://www.ncbi.nlm.nih.gov/COG/), the Gene ontology database (GO) (E-value < 1.0e^−5^) by BLASTX, and the NCBI nucleotide database (NT) (E-value < 1.0e^−5^) by BLASTN. Of the 67,873 unigenes, 50,548 were mapped at least in one public database. Of these being annotated unigenes, 47,045 were found in the NR database, 47,004 in the NT database, 28,465 in the Swiss-Prot database, 25,363 in KEGG database, 14,999 in COG database and 37,594 in the GO database (Table 3).

**Figure 2:**
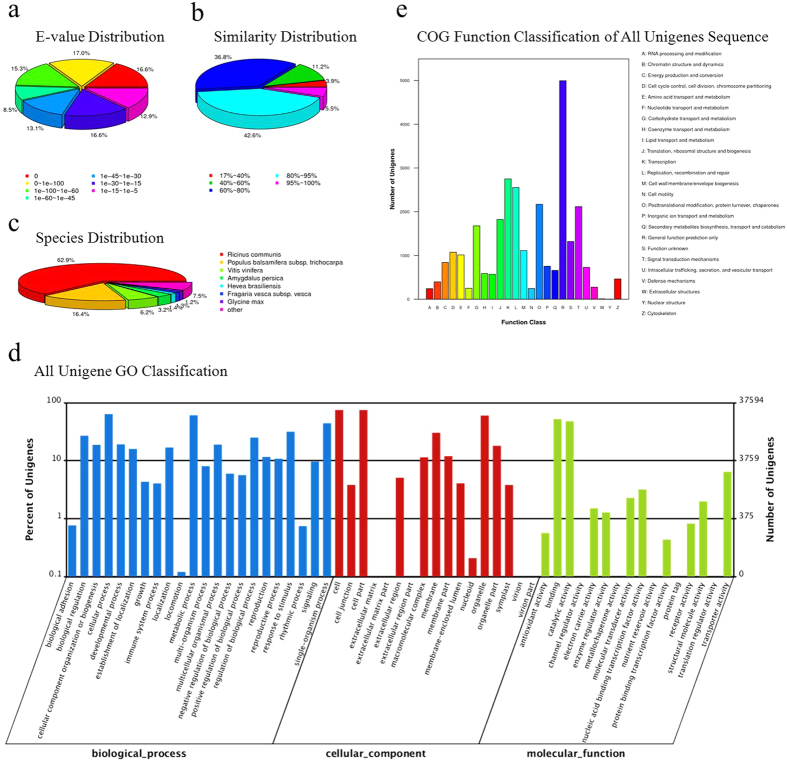
Annotation and classification of the all unigene set. (**a**) E-value distribution of the results from NR annotation. (**b**) Similarity distribution results from NR annotation. (**c**) Species distribution results from NR annotation. (**d**) GO classification analysis of the all unigene set. GO categories of ‘biological process’ (blue), ‘cellular component’ (red) and ‘molecular function’ (green) are shown on the X-axis. The right Y-axis shows the number of genes that have assigned GO functions, and the left Y-axis shows the percentage. (**e**) COG categories of all-unigenes.

According to the E-value frequency distribution from the annotation aligned to the NR database, 33,147 (70.46%) matched unigenes had strong homology with E-value < 1.0e^−30^ whereas 13,898 (29.54%) showed high similarity with the E-value from 1.0e^−30^ to 1.0e^−5^ (Fig. 2a). Analysis of the similarity distribution indicated that 22,607 (48.05%) unigenes shared more than 80% similarity with the matched genes (Fig. 2b). The unigenes were mainly matched to the sequences of *Ricinus communis* (29,606; 62.93% of 47,045) and *Populus balsamifera* subsp. *trichocarpa* (7,707; 16.38% of 47,045) (Fig. 2c). Using the Blast2GO program, a total of 37,594 unigenes were mapped and clustered into cellular component, biological process and molecular function categories. The top two subcategories were the ‘cell’ (28,103) and ‘cell part’ (28,101) in cellular component category, the ‘cellular process’ (23,864) and ‘metabolic process’ (22,818) in biological process category, and the ‘binding’ (19,618) and ‘catalytic activity’ (18,025) in molecular function category (Fig. 2d). The results of COG analysis showed that the matched 14,999 unigenes were clustered into 25 categories based on sequence homology (Fig. 2e). Of these 25 categories, the top five categories were the ‘General function prediction only’ (4,994), ‘Transcription’ (2,750), ‘Replication, recombination and repair’ (2,554), ‘Post translational modification, protein turnover, chaperones’ (2,168) and ‘Signal transduction mechanisms’ (2,118). By contrast, there were only 4 and 10 unigenes in the ‘Nuclear structure’ and ‘Extracellular structures’ categories, respectively (Fig. 2e). KEGG analysis showed that the matched 25,363 unigenes were assigned into 128 KEGG pathways (Supplementary Table S1). The top five enriched pathways were ‘Metabolic pathways’ (5,225), ‘Biosynthesis of secondary metabolites’ (2,386), ‘Plant-pathogen interaction’ (1,592), ‘Plant hormone signal transduction’ (1,517) and ‘Spliceosome’ (1,000).

### Differential transcriptomes between the COR and water treatments

**Figure 3:**
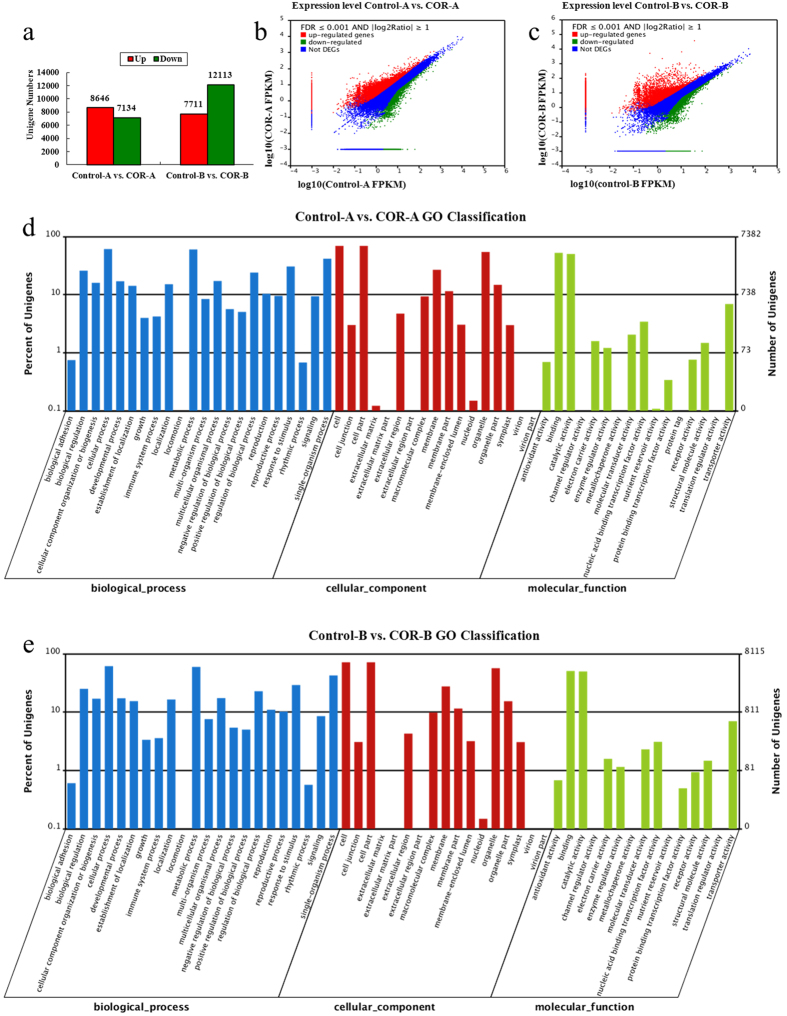
Statistical analysis and classification of differentially expressed unigenes. (**a**) Numbers of differentially expressed unigenes between COR and water treatments. (**b,c**) Distribution of differentially expressed genes (FDR ≤ 0.001 and |log2 ratio| ≥1). (**d,e**) GO classification analysis of the differentially expressed unigenes. GO categories of ‘biological process’ (blue)’, ‘cellular component’ (red) and ‘molecular function’ (green) are shown on the X-axis. The right Y-axis shows the number of genes that have identified GO functions, and the left Y-axis shows the percentage.

The differentially expressed genes (DEGs) were screened with the criteria of FDR ≤ 0.001 and |log2 ratio| ≥ 1 from transcriptome data comparison of Control-A vs. COR-A in the early response and Control-B vs. COR-B in the late response to COR and water treatments. A total of 8,646 unigenes were up-regulated whereas 7,134 unigenes were down-regulated in the early response to COR (Fig. 3a–c). In the late response to COR, 7,711 unigenes were up-regulated while 12,113 unigenes were down-regulated (Fig. 3a–c).

Using the Blast2GO program, the DEGs in the early response to COR were mainly enriched in ‘cell’ (5,102) and ‘cell part’ (5,102) subcategories of the ‘cellular component’ category, in the ‘cellular process’ (4,503) and ‘metabolic process’ (4,422) subcategories of the ‘biological process’ category, and in ‘binding’ (3,864) and ‘catalytic activity’ (3,697) subcategories of the ‘molecular function’ category (Fig. 3d). The DEGs in the late response to COR were also enriched in ‘cell’ (5,795), ‘cell part’ (5,795), ‘cellular process’ (4,974), ‘metabolic process’ (4831), ‘binding’ (4,112) and ‘catalytic activity’ (4,031) subcategories (Fig. 3e).

Analysis of GO functional enrichment of the DEGs was performed with a hypergeometric test (Bonferroni-correction *P* ≤ 0.05). The DEGs in the early response to COR were enriched in 8 GO terms while the DEGs in the late response to COR were enriched in 5 GO terms (Supplementary Table S2). The enriched 8 GO terms were ‘ADP binding’ (76), ‘methionine adenosyltransferase activity’ (14), ‘oxidoreductase activity, acting on paired donors, with incorporation or reduction of molecular oxygen’ (152) and ‘dioxygenase activity’ (80) in the category of molecular function, and ‘phenylpropanoid metabolic process’ (113), ‘S-adenosylmethionine biosynthetic process’ (14), ‘cellular modified amino acid metabolic process’ (70) and ‘phenylpropanoid biosynthetic process’ (83) in the category of biological processes. By contrast, the enriched 5 GO terms were ‘catalytic activity’ (4031), ‘oxidoreductase activity, acting on paired donors, with incorporation or reduction of molecular oxygen’ (164), ‘drug transporter activity’ (63), ‘transferase activity, transferring phosphorus-containing groups’ (1013) and ‘indole-3-acetic acid amido synthetase activity’ (9) in the category of molecular function.

A total of 4,820 unigenes of the DEGs in the early response to COR were assigned to 125 KEGG pathways. The top five enriched pathways were ‘Metabolic pathways’ (1,073), ‘Biosynthesis of secondary metabolites’ (546), ‘Plant-pathogen interaction’ (350), ‘Plant hormone signal transduction’ (304) and ‘Spliceosome’ (178) (Supplementary Table S3). Similarly, a total of 5,283 unigenes of the DEGs in the late response to COR were annotated using the KEGG database and were assigned to 125 KEGG pathways. The top five enriched pathways were also ‘Metabolic pathways’ (1,108), ‘Biosynthesis of secondary metabolites’ (562), ‘Plant-pathogen interaction’ (328), ‘Plant hormone signal transduction’ (294) and ‘Spliceosome’ (196) (Supplementary Table S4). The common DEGs are 634 in the DEGs of the top five biochemical pathways between the comparisons of Control-A vs. COR-A and Control-B vs. COR-B.

To experimentally validate the digital gene expression of the unigenes in transcriptome data, forty unigenes were randomly selected for qRT-PCR analysis. The expression patterns of most of the unigenes analysed by qRT-PCR were in accordance with the digital gene expression profiles (Supplementary Fig. S2), although the fold-changes obtained by qRT-PCR differed slightly from those obtained by the analysis of digital gene expression.

### Identification of the DEGs involved in JA signalling

**Figure 4:**
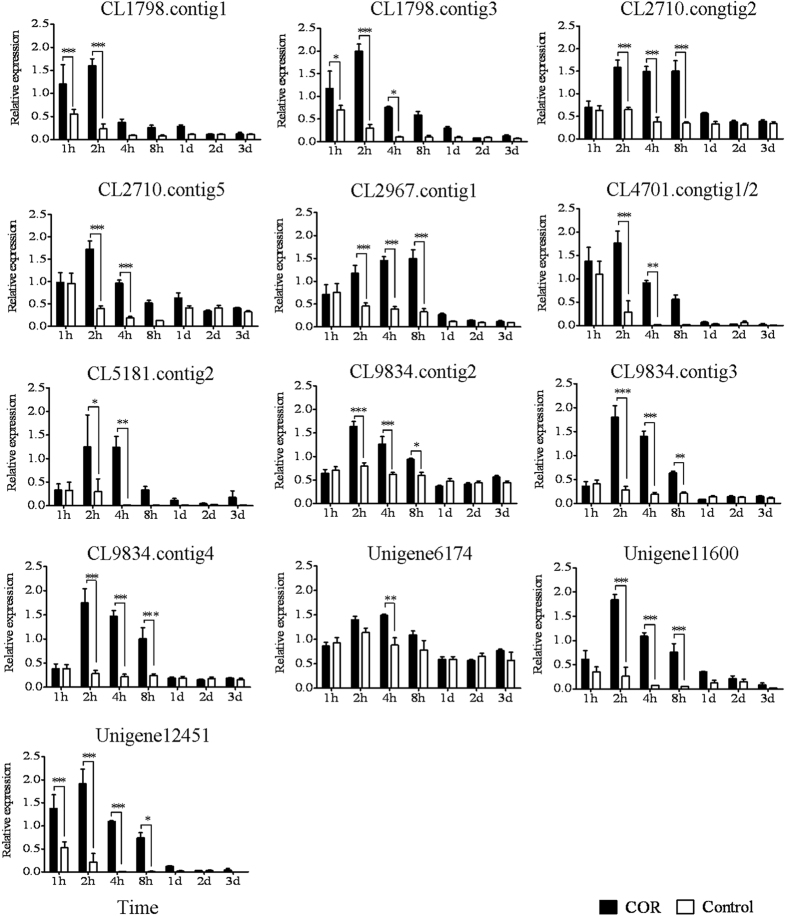
The qRT-PCR analysis of *JAZ* family members at different time intervals after COR and water treatments. The epicormic shoots were treated with either 20 μM COR or water. Cambia-containing tissues were collected at one hour (1 h), two hours (2 h), four hours (4 h), eight hours (8 h), one day (1 d), two days (2 d) and three days (3 d) after treatment. The relative expression of each gene was calculated as the 2^−ΔΔCt^ value and normalized to the endogenous reference gene, *HbUBC2a*. SD of three biological replicates is indicated by 1, 2 or 3 asterisks depending on the *P* value for the significant difference (*P* < 0.05, *P* < 0.01, *P* < 0.001, respectively) as determined by a one-way ANOVA.

**Figure 5:**
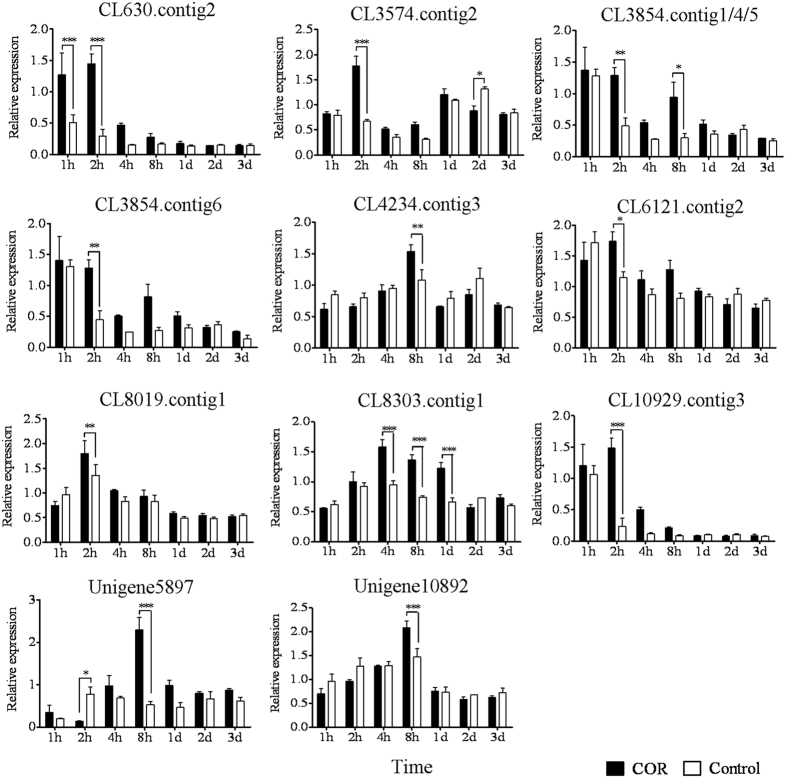
The qRT-PCR analysis of *MYC* family members at different time intervals after COR and water treatments. The materials and methods are as indicated in Fig. 4.

JA is a key signalling molecule in secondary laticifer differentiation^7^, which suggests that JA signalling pathway plays an important role in secondary laticifer differentiation. Jasmonate ZIM-domain protein (JAZ) and myelocytomatosis protein (MYC) have been identified as the core components of the JA signalling pathway in *Arabidopsis thaliana*^18^,^19^,^20^. There were 25 and 6 differentially expressed *JAZ* homologues in the Control-A vs. COR-A and Control-B vs. COR-B comparisons, respectively. In the Control-A vs. COR-A DEGs, almost all of the *JAZ* unigenes were significant up-regulated more than 2-fold (log2 ratio (COR-A/control-A) ≥ 1) except CL1965.Contig2 (Supplementary Table S5). Of these DEGs, the expression patterns of thirteen *JAZ* unigenes were validated by qRT-PCR (Fig. 4). The differential expression patterns of most unigenes were consistent with the results of the transcriptome analysis. Moreover, the qRT-PCR results showed that most of unigenes were significantly up-regulated and peaked at 2 h, and thereafter, they were down-regulated or remained relatively stable until 8 h after COR treatment (Fig. 4). A total of 45 differentially expressed *MYC* homologues were identified. Of these, 31 and 22 corresponding unigenes were detected, respectively, in the Control-A vs. COR-A and the Control-B vs. COR-B DEGs. Of these, 8 unigenes were differentially expressed in both comparisons. At the early stage (from 1 h to 4 h) of response to COR, most *MYC* unigenes (25) were up-regulated more than 2-fold (log2 ratio (COR-A/control-A) ≥ 1) (Supplementary Table S5). However, at the late stage (from 8 h to 3 d), most *MYC* unigenes (18) were down-regulated more than 2-fold (log2 ratio (COR-B/control-B) ≤ -1) in response to COR. Subsequently, the expression patterns of eleven *MYC* unigenes were validated by qRT-PCR. The results indicated that these *MYC* DEGs were significantly up-regulated following COR treatment. Of the differentially expressed *MYC* unigenes, seven peaked at 2 h and three peaked at 8 h following COR treatment. There was only one unigene (CL8303.contig1) that peaked at 4 h after COR treatment (Fig. 5). The digital gene expression data and qRT-PCR validation indicated that the differentially expressed unigenes of *JAZ* and *MYC* homologues were mainly up-regulated at the early stage of response to COR.

### Identification of the DEGs involved in the CLAVATA (CLV) signalling pathway

**Figure 6:**
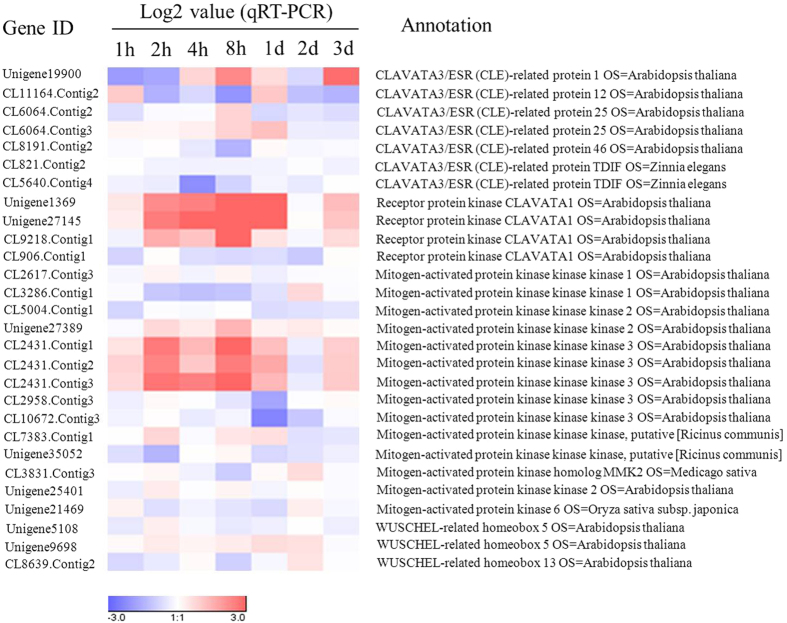
The qRT-PCR analysis of DEGs involved in the CLAVATA-MAPK-WOX signalling pathway. Gene expression Log 2 values displayed as a heat-map. The values were normalized using *HbUBC2a* as an internal control, and the ratio of COR-treated sample to control sample has been calculated for each time point. A color bar represents the expression level of each gene with red for up-regulated and blue for down-regulated.

Available data show that CLAVATA (CLV) signalling pathway plays a crucial role in regulating the homeostasis of stem cell proliferation and differentiation^21^,^22^. The key components of the CLV signalling pathway are the CLV3/Embryo Surrounding Region-related (CLE) peptides, CLV1 and WUSCHEL (WUS) or WUS-related homeobox (WOX)^23^,^24^,^25^,^26^. Mitogen-activated protein kinase (MAPK) cascades may mediate the CLV signalling pathway downstream of CLV receptors^27^,^28^. Twenty-eight DEGs that are related to the CLV-MAPK-WOX signalling system were identified and analyzed by qRT-PCR (Fig. 6, Supplementary Table S6 and Supplementary Table S7). The unigenes encoding CLE-related proteins (unigene19900, CL11164.Contig2 and CL6064.Contig3) and CLV1 (unigene1369, unigene27145 and CL9218.Contig1) were significantly up-regulated more than 2-fold (log2 value ≥ 1). Specifically, unigene19900 (CLE-related protein 1) was down-regulated at 1 h and 2 h, followed by up-regulation at 4 h, and it reached a peak at 8 h after COR treatment. The CL6064.Contig3 (CLE-related protein 1) was up-regulated from 1 h to 1 d. It is noteworthy that unigene1369 and unigene27145 (receptor protein kinase CLV1) were significantly up-regulated from 1 h to 1 d, with more than 20-fold up-regulation at 8 h after COR treatment. The unigenes involved in the MAPK cascades, that were up-regulated more than 2-fold in response to COR included unigene27389 (Mitogen-activated protein kinase kinase kinase 2, MAPKKK2) and CL2431.Contig1/2/3 (MAPKKK3). It is also noteworthy that the relative expression levels of CL2431.Contig1 and CL2431.Contig3 were significantly up-regulated more than 10-fold at 8 h. By contrast, some MAPKKK family-related unigenes such as CL3286.Contig1, CL2958.Contig3, CL10672.Contig3 and unigene35052 were down-regulated more than 2-fold (Log2 value ≤ −1) at various time intervals in response to COR. The WOX-related unigene5108 and CL8639.Contig2 were also significantly down-regulated within 8 h in response to COR (Fig. 6 and Supplementary Table S7).

## Discussion

**Figure 7:**
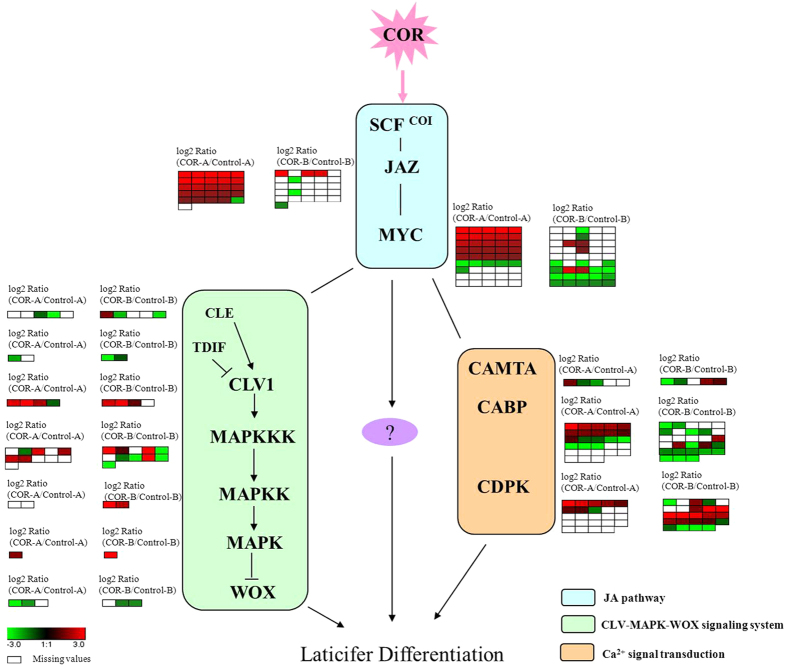
Signalling networks in regulating the secondary laticifer differentiation from vascular cambia in rubber trees. The heatmap shows Log 2 values of the digital gene expression in transcriptome. A colour square represents the expression level of each gene that was either up-regulated (red) or down-regulated (green) in response to COR. A blank square represents missing expression values in the group.

For rubber tree de novo assembly, approximately 5–8 Gb of reads ensures approximately 90% transcriptome coverage with optimized k-mers and transcript N50 length^29^. In this study, we achieved more than 7 Gb of clean reads from each of the four sets of sequencing samples derived from the cambia-containing materials of rubber tree clone CATAS7-33-97. The sequencing depth of each sample was higher than the reported 0.2–6 Gb sequencing depth of bark in rubber tree^30^,^31^,^32^,^33^,^34^ and equivalent to sequencing depth (7–8 Gb) reported by Mantello *et al.*^35^. This high sequencing depth is beneficial for global transcriptome assembly. In the present study, a total of approximately 30 Gb of clean reads were de novo assembled, which generated 67,873 unigenes. The number of unigenes is close to the 68,955 gene models predicted in the draft genome sequence by Rahman *et al.*^36^, and falls in the range of the 43,792 to 84,440 predicted protein-coding genes in the rubber tree genome^37^,^38^. To our knowledge, this is the first report of transcriptome data illustrating the response of the cambia-containing materials to COR treatment. Therefore, the high quality of the assembly data in the present study extends the rubber tree bark transcriptome database and will be helpful for revealing the regulatory networks involved in the differentiation of secondary laticifers from vascular cambia. In this study, the digital gene expression of the randomly selected forty genes is in accordance with the qRT-PCR validation. And the digital gene expression is up-regulated for most of *JAZ* and *MYC* unigenes (Fig. 7), and available data show that the genes of *JAZ* and *MYC* families are generally up-regulated by jasmonates^39^,^40^. The COR is more effective in inducing the secondary laticifer differentiation at lower concentration and within shorter time in comparison with jasmonates^7^. Moreover, the cell division in the region of vascular cambia was observed three days after JA treatment^7^. Therefore, the differentially expressed genes within three days should be related the COR-induced laticifer differentiation.

JAZs and MYCs are the key components of JA signalling pathway^18^,^19^,^20^. The JAZ proteins are characterized by the presence of ZIM and Jas domains and their interaction with COI1 in the presence of JA-Ile^41^,^42^. There are 13 JAZ proteins in *Arabidopsis thaliana*^20^,^43^,^44^,^45^. Available data indicated that AtJAZ1, AtJAZ3 and AtJAZ9 interacted with COI1^41^,^42^,^46^ and AtJAZ1-6 and AtJAZ8-13 interacted with MYC2^45^,^46^,^47^. These JAZ members mediated JA response^20^,^43^,^48^. Although several *JAZ* homologues were isolated from the latex of rubber trees^49^,^50^,^51^, little is known about their roles in secondary laticifer differentiation. In the present study, 5 of the 13 qRT-PCR validated JAZ homologous unigenes have complete open reading frame (ORF) with ZIM and Jas domains whereas the others’ ORF were incomplete (NCBI’ GEO accession numbers: GSE80596). Some of these JAZ homologues were annotated as AtJAZ1 (CL1798.contig3 and unigene12541), AtJAZ2 (CL1798.contig1), AtJAZ3 (CL2710.contig2/5 and CL9834.contig3/4), AtJAZ8 (Unigene11600), AtJAZ10 (CL5181.contig2) and AtJAZ12 (CL2967.contig1) (Supplementary Table S5). These JAZ homologues may be the members of JAZ components of JA signalling pathway and mediated COR-induced secondary laticifer differentiation in rubber trees. The MYC proteins are a kind of transcription factors of bHLH family^52^. The AtMYC2 was one of the most intensively demonstrated MYC members in JA signalling pathway^25^. In addition, AtMYC6 (also known as GLABRA3) plays a role in tricome development and epidermal cell fate in *Arabidopsis*^53^,^54^ and AtbHLH13 negatively regulates JA-mediated plant defense and development in a COI1-dependent manner^55^. In the present study, some of the qRT-PCR validated MYC homologous unigenes were annotated as AtMYC2 (CL3854.contig1/4/5/6), AtMYC6 (CL4234.contig3 and CL8303.contig1) and AtbHLH13 (CL630.contig2) (Supplementary Table S5). These MYC homologues may be the members of MYC components of JA signalling pathway and mediated COR-induced secondary laticifer differentiation in rubber trees.

Owing to the complexity of cell differentiation, the differentially expressed genes that are related to other signalling pathways may be involved in secondary laticifer differentiation at downstream of the JA pathway or via cross-talk with the JA pathway. The unigenes encoding the components of Ca^2+^ signal transduction, such as *CAMTA* (calmodulin-binding transcription activator), *CDPK* (calcium-dependent protein kinase) and *CABP* (calmodulin binding protein) which may involved in laticifer differentiation^16^, were also found to be differentially expressed in the transcriptome (Fig. 7, Supplementary Table S8). The CLAVATA (CLV) signalling pathway and MAPK cascades may participate in the regulation of JA signalling for secondary laticifer differentiation. The CLV signalling pathway is known to play a key role in stem cell homeostasis^21^,^22^ and is involved in plant immune responses^56^,^57^. CLV1 encodes a transmembrane receptor kinase and plays a key role in the CLV pathway by binding to the CLE peptide. The CLV3/CLE-CLV1 ligand-receptor kinase pair has a role in negatively regulating the WUSCHEL (WUS) transcription factor whereas WUS promotes CLV3 expression^26^. In the present study, four unigenes (unigene27145, unigene1369, CL9218.Contig1 and CL906.Contig1) encoding CLV1 were differentially expressed between the COR- and water-treated samples. Unigene27145, unigene1369 and CL9218.Contig1 were all up-regulated more than 2-fold (log2 ratio ≥ 1) in both the early and late responses to COR, whereas CL906.Contig1 was down-regulated in the early response to COR (Fig. 7). The qRT-PCR results showed that unigene27145, unigene1369 and CL9218.Contig1 were significantly up-regulated at 2 h and peaked at 8 h after COR treatment. Of these unigenes, unigene27145 and unigene1369 were up-regulated more than 20-fold at 8 h after COR treatment compared to the control (Supplementary Table S7, Fig. 6). The available data show that MAPK cascades mediate JA signalling and the negative regulation of WUS by the CLV signalling. In *Arabidopsis*, MYC2 interacts with the promoter of *AtMPK6* and regulates its expression, while MYC2 is also phosphorylated by the MKK3-MPK6 module^58^,^59^. AtMPK6, a potential downstream target of CLV signalling, is activated by the CLV3 stimuli in *Arabidopsis* and in *Nicotiana benthamiana*^27^. This activated MPK6 may suppress WUS expression and play a role in regulating the homeostasis of the shoot apical meristem (SAM)^27^. Generally, MAPK cascades consist of three components: MAPK kinase kinase (MAPKKK), MAPK kinase (MAPKK) and MAPK. In the studied transcriptome, more than ten DEGs encoding MAPKKKs were detected. Some members such as CL5004.Contig1, CL2431.Contig1, CL2431.Contig2 and CL2431.Contig3 were significantly up-regulated in the early response to COR, while other members such as CL2617.Contig3, Unigene27389, CL10672.Contig3 and CL3286.Contig1 were significantly up-regulated in the late response to COR (Fig. 7). The qRT-PCR analysis confirmed that most of the *MAPKKK* unigenes had expression patterns similar to the digital gene expression data (Figs 6 and 7). Of these unigenes, CL2431.Contig1and CL2431.Contig3 which are annotated as MAPKKK3 were up-regulated sharply more than 10-fold at 8 h after COR treatment (Supplementary Table S7, Fig. 6). Unigene25401 encoding MAPKK2 was up-regulated at 2 h and 8 h after COR treatment (Fig. 6). Unigene21469, which is annotated to be MPK6, was up-regulated at 2 d after COR treatment (Fig. 6). By contrast, the unigenes (unigene5108 and CL8639.contig2) encoding WOX, key regulators of stem cell fate in different meristems^60^, were significantly down-regulated at 8 h after COR treatement (Fig. 6).

Taken together, the findings in the present study indicated a relationship between JA signalling, the CLV-MAPK-WOX signalling pathway and Ca^2+^ signal transduction in the regulation of COR-induced secondary laticifer differentiation in rubber trees (Fig. 7).

## Methods

### Plant materials

Plantlets of rubber tree (*Hevea brasiliensis* Muell. Arg.) clone CATAS7-33-97, budded on rootstocks, were grown at the Experimental Station of the Rubber Research Institute of the Chinese Academy of Tropical Agricultural Sciences (RRI-CATAS) in Danzhou city, Hainan province, P.R. China. The plantlets were pruned each year, and epicormic shoots grew from the latent buds on the pruned branches. Each epicormic shoot flushes five to six times a year and includes a series of foliage clusters which are separated by the lengths of the leafless stems. Therefore, each of these morphologically distinct growth increments represents a growth flush and is referred to as an extension unit (EU)^7^,^11^. Under natural conditions, no secondary laticifers appear in the stem of EU1-2 (counted from the top of the shoot)^7^,^11^, which is convenient for distinguishing the induced secondary laticifers. In this study, treatments were performed on the stem of EU2 to collect enough cambia-containing inner bark samples for total RNA extraction.

### Experimental design and sample preparation

The epidermis and part of the cortex (within an area of 2 cm × 4 cm in the middle of the EU2 stem) were removed by scratching with a single sided razor blade. The wounded surface was immediately wrapped in a polyethylene membrane after being treated with 20 μM COR (Sigma, USA) or sterile water^16^. The vascular cambia-containing samples were obtained by collecting the materials that were scratched from the inner surface of the peeled bark and the outer surface of the exposed xylem after the treated bark had been peeled off (Supplementary Fig. S3). The samples collected at one hour (1 h), two hours (2 h), four hours (4 h), eight hours (8 h), 1 day (1 d), 2 days (2 d) and 3 days (3 d) after treatments. Samples from nine epicormic shoots were collected for each time interval, immediately frozen in liquid nitrogen and stored at −80 °C until RNA extraction. The samples for each time interval were a mixture of material from the nine epicormic shoots. In this way, a total of 14 mixed samples were obtained for RNA isolation.

For paraffin-embedded sections of the secondary laticifer differentiation induced by COR, the epidermis and outer parts of the cortex within an area of 0.2 cm × 1 cm were scratched with a one-sided blade in the middle of the EU2 stem^11^. The wounded surface of each of the five epicormic shoots was wrapped immediately with parafilm membrane after with the application of either 20 μM COR or sterile water^16^.

For quantitative real-time RT-PCR (qRT-PCR) analysis, total RNA was extracted from each of the nine epicormic shoots at each time interval. The total RNA from three shoots was equivalently pooled to produce three biological replicates at each time interval.

### RNA isolation and Illumina sequencing

Total RNA was isolated separately from the 14 mixed samples according to the procedure of Tian *et al.*^61^. A NanoDrop ND-2000 spectrophotometer (Thermo Fisher Scientific, USA) and Agilent 2100 Bioanalyzer (Agilent, USA) were used to determine the total RNA concentration and quality. For RNA-seq, the seven total RNA samples from the 1 h, 2 h and 4 h time intervals and the 8 h, 1 d, 2 d and 3 d time intervals following COR treatment were pooled in equivalent amounts to generate a mixed RNA sample of early response stage and a mixed RNA sample of late response stage, respectively. The seven total RNA samples from the water treatments were mixed in the same manner. Then, the four mixed RNA samples were used to construct four paired-end (PE) cDNA libraries with an average insert size of 200 bp. The two cDNA libraries for the early response stage are referred to as ‘Control-A’ for the water treatment and ‘COR-A’ for the COR treatment. The two cDNA libraries for the late response stage are respectively referred to as ‘Control-B’ for the water treatment and ‘COR-B’ for the COR treatment. The four cDNA libraries were separately sequenced using the Illumina HiSeq^TM^ 2000 (Illumina Inc., San Diego, CA, USA) in PE90 model.

### Light microscopy

The bark samples were collected 5 days after treatment and fixed in 80% ethanol for 24 h at room temperature to eliminate tannin-like substances that may be mistaken for rubber inclusions in laticifers^7^. Next, the samples were treated with iodine and bromine in glacial acetic acid and embedded in paraffin after dehydration. Sections (12 μm in thickness) were cut with a microtome (Leica Microsystems Inc., Bannockburn, IL, Germany) and stained with fast green dye. The laticifers in sections were recognized by brown color of the rubber as a result of iodine-bromine treatment.

### De novo assembly, annotation and classification of the transcriptome

The de novo assembly, annotation and classification of the transcriptome were performed at BGI (BGI, Shenzhen, China), as described in previous reports^62^. The raw reads were initially generated by sequencing with an Illumina HiSeq^TM^ 2000 platform. Before assembly, adaptor sequences, empty reads, low quality sequences with ‘N’ percentage over 5% and those containing more than 20% bases with a Q-value < 10 were removed using the in-house program Filter_fq written in Perl according to the custom method of program editing. Then, the clean reads were used for de novo assembly. De novo assembly was carried out using SOAPdenovo (http://soap.genomics.org.cn/soapdenovo.html) with default parameters (BGI, Shenzhen, China). Overlapping reads were combined into contigs by using SOAPdenovo with an adjusted K-mer value (K = 25). Subsequently, adjacent contigs were connected into scaffolds using ‘N’ to represent unknown bases and insert size information. Finally, the extended sequences with the least Ns, which were defined as unigenes, were generated by scaffold gap filling with paired-end information. The “all unigenes” was assembled from the unigenes of Control-A, COR-A, Control-B and COR-B and used as a reference transcriptome for further analyses.

To obtain annotation information on the inner-bark transcriptome in rubber tree, the sequences of the all unigenes were aligned with the nucleotide database NT (E-value<1.0e^−5^) by BLASTN and with the protein databases (including NR (NCBI non-redundant protein sequences), Swiss-Prot (http://www.expasy.ch/sprot/), KEGG (http://www.genome.jp/kegg/) and COG (http://www.ncbi.nlm.nih.gov/cog/) (E-value of 1.0e^−5^) by BLASTX. The annotation assignment from the protein databases was prioritized as NR, NT, Swiss-Prot, KEGG and COG. For classification of the transcriptome, all of the unigenes were assigned to three gene ontology (GO) categories (molecular function, biological process, and cellular component) using the Blast2GO software (http://www.geneontology.org)^63^. The GO functional enrichment analysis was performed to find significantly enriched GO terms from DEGs using a hypergeometric test (Bonferroni-correction *P* ≤ 0.05) compared the reference transcriptome. The unigenes were mapped to metabolic pathways using KEGG.

### Digital gene expression profiling

According to the methods described by Audic *et al.*^64^, a rigorous algorithm was developed to identify the differentially expressed genes (DEGs) between COR and water treatments. In the present study, the FPKM (Fragments Per Kilobase of transcripts per Million mapped reads) method^65^ was used to calculate the expression levels of all unigenes. Specifically, the expression level was calculated using the formula FPKM = 10^9^ × C/(N × L), where C is the number of reads aligned to a target unigene, N is the total number of reads aligned to all of the unigenes, and L is the base number of the target unigene. The assembled transcriptome was used as a reference database. The target unigenes with a value of the log2 (FPKM_COR_/FPKM_Control_) ≥1 and an FDR (false discovery rate) ≤0.001 were considered DEGs between the COR and water treatments. Twenty genes were randomly selected from the DEGs in the comparison of Control-A vs. COR-A and twenty genes were randomly selected from the DEGs in the comparison of Control-B vs. COR-B to validate the digital gene expression profiles in Control-A vs. COR-A and Control-B vs. COR-B by qRT-PCR, respectively (Fig. S2; Table S6).

### Quantitative real-time RT-PCR analysis

Approximately 1 μg of the mixed total RNA samples from every three shoots was treated with DNase I and thereafter was reversely transcribed according to the manufacturer’s instructions of RevertAid™ First Strand cDNA Synthesis Kit (Thermo Scientific Inc., USA) with the oligo dT18 primer. The first strand cDNA was diluted 20-fold and used as the template for qRT-PCR analysis. The qRT-PCR was performed in a 10 μl reaction volume using SYBR Premix Ex Taq™ (Tli RNase H Plus) (TaKaRa, Japan) in CFX384 Real-Time PCR Detection System (Bio-Rad Laboratories, Inc., USA). Three technical replicates were assayed for each reaction. The procedure for qRT-PCR was as follows: 1 min at 95 °C for denaturation, followed by 45 cycles of 95 °C for 10 s, 60 °C for 20 s and 72 °C for 20 s. Then, and a melt curve analysis of amplified products was made. Twenty-two housekeeping genes (*Hb18S, HbActin, HbADF, HbADF4, HbCYP2, HbeIF1Aa, HbeIF1Ab, HbeIF2, HbeIF3, HbFP, HbPTP, HbRH2a, HbRH2b, HbRH8, HbROC3, HbTCBP, HbUBC1, HbUBC2a, HbUBC2b, HbUBC3, HbUBC4, HbYLS8*)^66^ were screened to select a suitable reference gene using geNorm^67^ and the software package NormFinder (version 0.953, http://www.mdl.dk/publicationsnormfinder.htm). Of these, *HbUBC2a* was the most stable (Supplementary Fig. S4) and therefore was used as the endogenous reference gene for template normalization in this study. The relative expression values of the selected genes were calculated using the 2^−ΔΔCT^ method^68^ in the CFX384 Real-Time PCR Detection System. All the primers were manufactured by Invitrogen (Shanghai, China) (Supplementary Table S9). The statistical significance of the difference in the relative transcript abundances between COR and water treatments was analysed by one-way ANOVA.

## Additional Information

**Accession codes:** The assembled sequences and sequencing reads were submitted to the NCBI’s Gene Expression Omnibus (GEO) repository and the data are available at the following URL: http://www.ncbi.nlm.nih.gov/geo/query/acc.cgi?acc=GSE80596.

**How to cite this article**: Wu, S. *et al.* Transcriptome Analysis of the Signalling Networks in Coronatine-Induced Secondary Laticifer Differentiation from Vascular Cambia in Rubber Trees. *Sci. Rep.* **6**, 36384; doi: 10.1038/srep36384 (2016).

**Publisher’s note**: Springer Nature remains neutral with regard to jurisdictional claims in published maps and institutional affiliations.

## Supplementary Material

Supplementary Information

## Figures and Tables

**Table 1:** Summary of output statistics from four RNA-seq samples treated with COR and Control, respectively.

Samples	Total Raw Reads	Total Clean Reads	Total Clean Nucleotides (nt)	Q20 percentage	N percentage	GC percentage
Control-A	87,101,818	80,829,264	7,274,633,760	98.00%	0.00%	44.35%
Control-B	85,745,464	79,834,296	7,185,086,640	98.02%	0.00%	43.83%
COR-A	86,766,790	81,412,500	7,327,125,000	98.39%	0.03%	44.08%
COR-B	87,936,166	81,766,122	7,358,950,980	98.22%	0.03%	42.10%

**Table 2:** Summary of assembly statistics from four RNA-seq samples treated with COR and Control, respectively.

Contig/Unigene	Sample	Total Number	Total Length(nt)	Mean Length(nt)	N50	Total Consensus Sequences	Distinct Clusters	Distinct Singletons
Contig	Control-A	130,199	41,187,179	316	467	—	—	—
	Control-B	143,435	43,022,396	300	444	—	—	—
	COR-A	121,094	30,134,860	249	301	—	—	—
	COR-B	131,686	28,961,041	220	239	—	—	—
Unigene	Control-A	75,468	40,631,229	538	915	75,468	25,308	50,160
	Control-B	71,994	42,879,697	596	1003	71,994	26,850	45,144
	COR-A	58,479	25,415,975	435	600	58,479	19,037	39,442
	COR-B	58,907	22,153,994	376	455	58,907	17,628	41,279
	All	67,873	50,932,972	750	1222	67,873	28,868	39,005

**Table 3:** Summary of annotation statistics of all unigenes.

Sequence	NR	NT	Swiss-Prot	KEGG	COG	GO	ALL
All Unigenes	47,045	47,004	28,465	25,363	14,999	37,594	50,548

NR (non-redundant database), NT (nucleotide database), Swiss-Prot (Swiss Protein database), KEGG (Kyoto Encyclopedia of Genes and Genomes), COG (Clusters of Orthologous Groups), GO (Gene Ontology).
