# Supplementary material for: Transcriptome Analysis of the Signalling Networks in Coronatine-Induced Secondary Laticifer Differentiation from Vascular Cambia in Rubber Trees
Source: Sci Rep. 2016 Nov 3;6:36384. doi: 10.1038/srep36384 (PMC5093416; doi:10.1038/srep36384)
Supplement: Supplementary Information [file srep36384-s1.pdf]

Supplementary Information

Transcriptome Analysis of the Signalling Networks in Coronatine-Induced Secondary Laticifer Differentiation from Vascular Cambia in Rubber Trees

Shaohua Wu, Shixin Zhang, Jinquan Chao, Xiaomin Deng, Yueyi Chen, Minjing Shi & Wei-Min Tian*

Ministry of Agriculture Key Laboratory of Biology and Genetic Resources of Rubber Tree / State Key Laboratory Breeding Base of Cultivation and Physiology for Tropical Crops, Rubber Research Institute, Chinese Academy of Tropical Agricultural Sciences, Danzhou, Hainan 571737, P.R. China

*Corresponding author: Email: wmtian@163.com, Tel: 86-898-23300309; Fax: 86-898-23300315

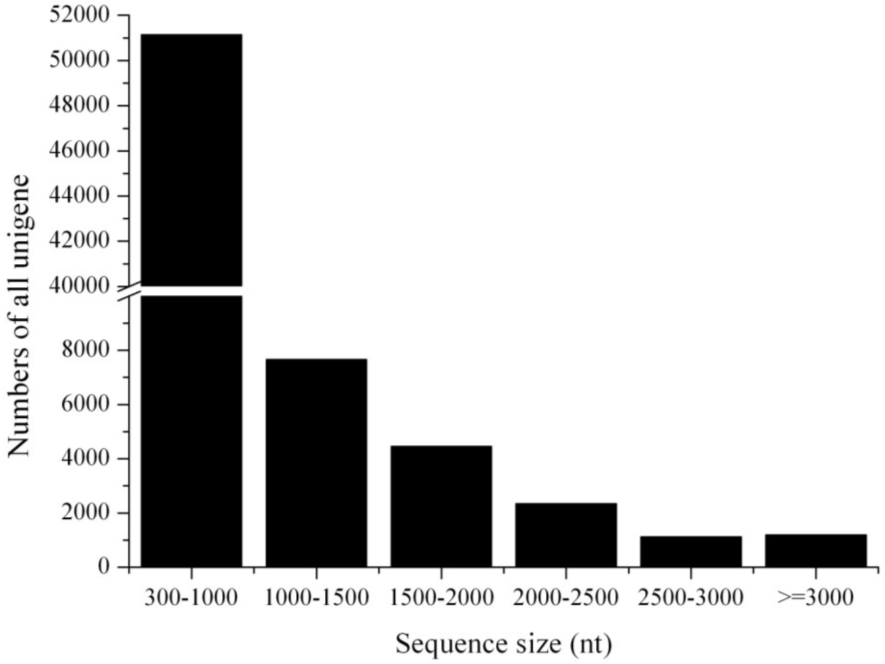

Supplementary Fig. S1. Size distribution of all unigene.

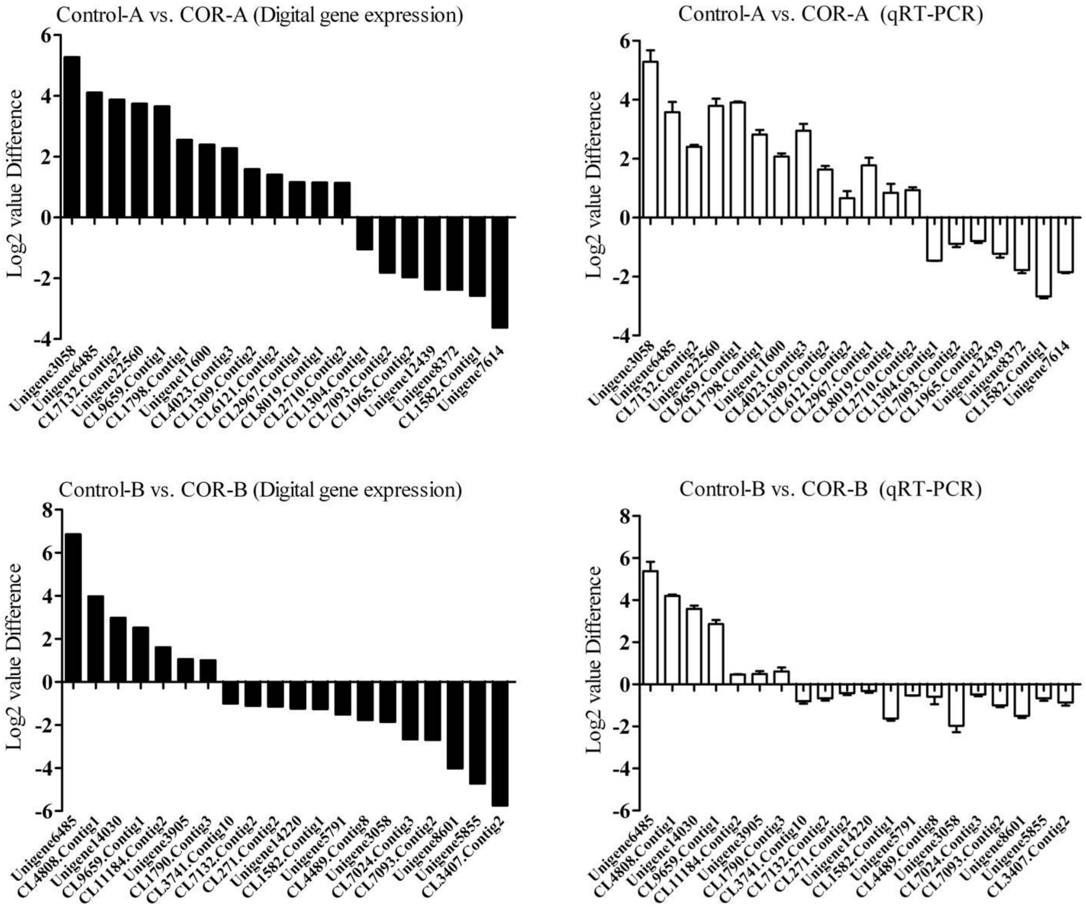

Supplementary Fig. S2. Validation of digital gene expression by qRT-PCR. The white column charts showed the expression profiles by qRT-PCR and the black column charts showed the digital gene expression profiles. Error bars for qRT-PCR showed the standard deviation (SD) of three replicates.

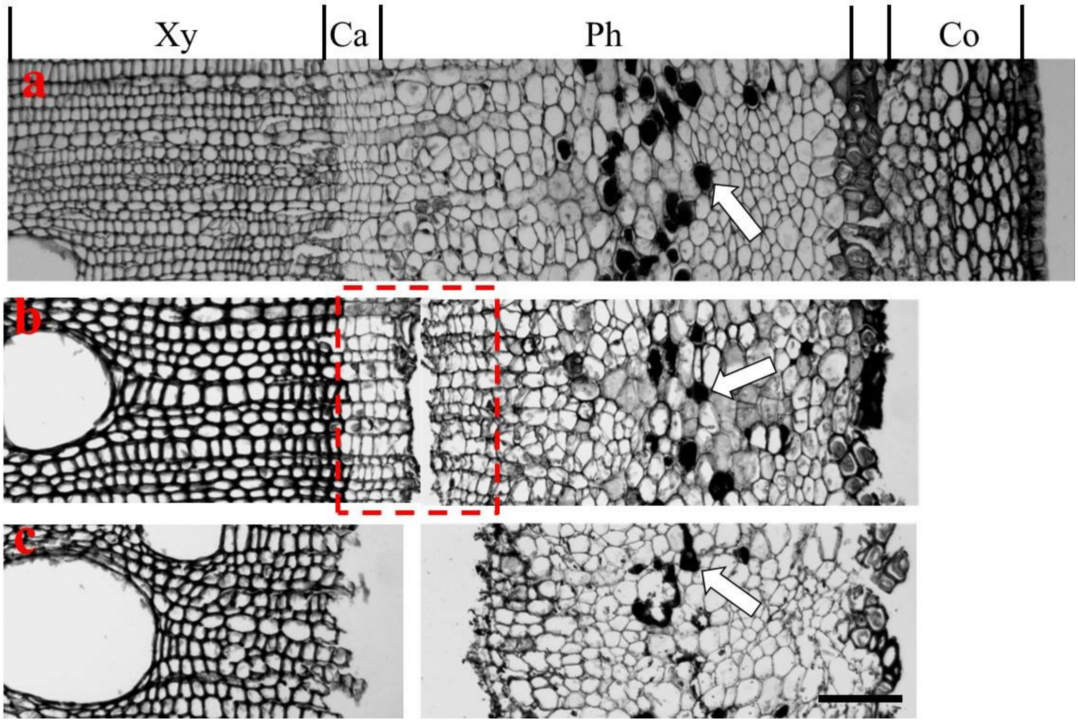

Supplementary Fig. S3. Light micrographs of stem cross-sections, showing the method and regions that the sample was collected. (a) the whole stem. (b) the separated xylem (left) and bark (right) by peeling. (c) the xylem (left) and bark (right) after being scratched the the outer surface of the exposed xylem and the inner surface of the separated bark. Red dotted box showed the collected cambia-containing sample. White arrows showed the primary laticifers. Xy, xylem; Ca, cambia; Ph, phloem; Co, cortex. Bars = 100 µm.

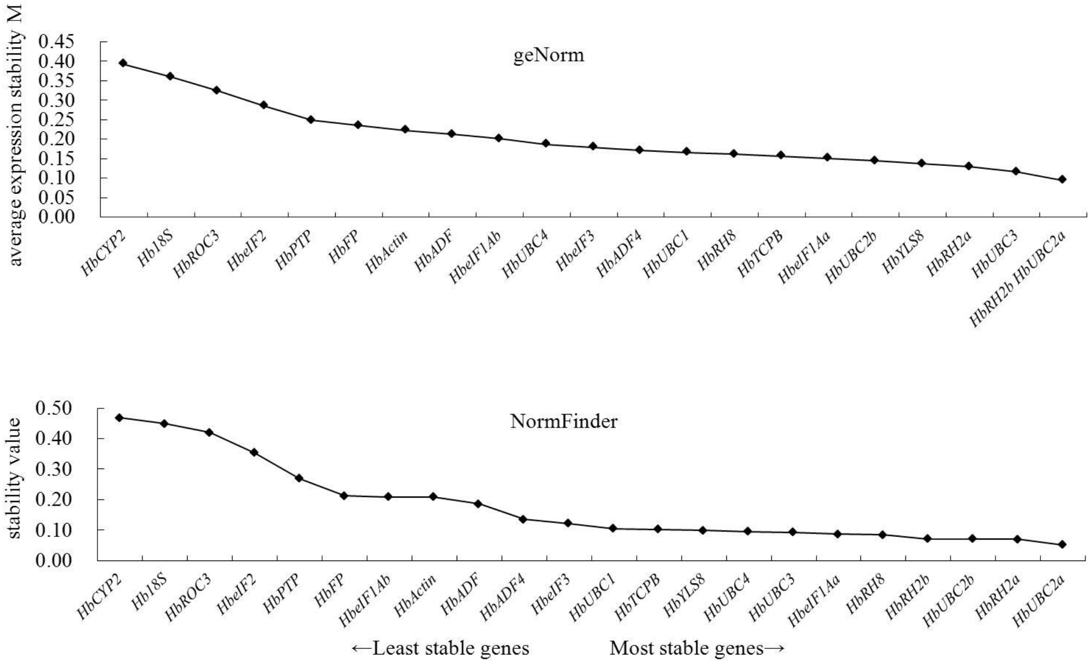

Supplementary Fig. S4. Evalution of the stability in the expression of 22 candidate reference genes in response to either water or COR treatment by geNorm and NormFinder analysis. The least stable genes are arranged on the left whereas the most stable genes are on the right.

Supplementary Table S1. KEGG pathways of all assembled unigenes

#	Pathway	Number (25363)	Pathway ID
1	Metabolic pathways	5225	ko01100
2	Biosynthesis of secondary metabolites	2386	ko01110
3	Plant-pathogen interaction	1592	ko04626
4	Plant hormone signal transduction	1517	ko04075
5	Spliceosome	1000	ko03040
6	RNA transport	960	ko03013
7	Protein processing in endoplasmic reticulum	713	ko04141
8	Purine metabolism	689	ko00230
9	Endocytosis	629	ko04144
10	Pyrimidine metabolism	621	ko00240
11	Starch and sucrose metabolism	580	ko00500
12	Ubiquitin mediated proteolysis	533	ko04120
13	Ribosome	530	ko03010
14	mRNA surveillance pathway	528	ko03015
15	Glycerophospholipid metabolism	528	ko00564
16	RNA degradation	502	ko03018
17	Ribosome biogenesis in eukaryotes	476	ko03008
18	RNA polymerase	403	ko03020
19	Ether lipid metabolism	387	ko00565
20	Oxidative phosphorylation	350	ko00190
21	Phenylpropanoid biosynthesis	348	ko00940
22	Amino sugar and nucleotide sugar metabolism	281	ko00520
23	Glycolysis / Gluconeogenesis	279	ko00010
24	Phagosome	271	ko04145
25	ABC transporters	255	ko02010
26	Circadian rhythm - plant	247	ko04712
27	Peroxisome	227	ko04146
28	Pentose and glucuronate interconversions	226	ko00040
29	Homologous recombination	221	ko03440
30	Cysteine and methionine metabolism	216	ko00270
31	Nucleotide excision repair	208	ko03420
32	Pyruvate metabolism	205	ko00620
33	Aminoacyl-tRNA biosynthesis	194	ko00970
34	Phosphatidylinositol signaling system	188	ko04070
35	Flavonoid biosynthesis	184	ko00941
36	Arginine and proline metabolism	181	ko00330
37	Inositol phosphate metabolism	175	ko00562
38	Galactose metabolism	171	ko00052
39	Basal transcription factors	167	ko03022
40	Phenylalanine metabolism	166	ko00360
41	Glycine, serine and threonine metabolism	165	ko00260

42	Stilbenoid, diarylheptanoid and gingerol biosynthesis	163	ko00945
43	Base excision repair	160	ko03410
44	Terpenoid backbone biosynthesis	158	ko00900
45	DNA replication	158	ko03030
46	Carbon fixation in photosynthetic organisms	155	ko00710
47	Glutathione metabolism	154	ko00480
48	Cyanoamino acid metabolism	152	ko00460
49	Glyoxylate and dicarboxylate metabolism	150	ko00630
50	Regulation of autophagy	147	ko04140
51	Glycosylphosphatidylinositol(GPI)-anchor biosynthesis	140	ko00563
52	Fructose and mannose metabolism	138	ko00051
53	Glycerolipid metabolism	133	ko00561
54	Protein export	131	ko03060
55	Other glycan degradation	130	ko00511
56	Zeatin biosynthesis	128	ko00908
57	Ascorbate and aldarate metabolism	127	ko00053
58	Nitrogen metabolism	126	ko00910
59	Limonene and pinene degradation	125	ko00903
60	SNARE interactions in vesicular transport	125	ko04130
61	Mismatch repair	124	ko03430
62	Valine, leucine and isoleucine degradation	122	ko00280
63	Carotenoid biosynthesis	122	ko00906
64	Proteasome	122	ko03050
65	Alanine, aspartate and glutamate metabolism	121	ko00250
66	Porphyrin and chlorophyll metabolism	120	ko00860
67	N-Glycan biosynthesis	118	ko00510
68	Pentose phosphate pathway	116	ko00030
69	Tyrosine metabolism	113	ko00350
70	Citrate cycle (TCA cycle)	111	ko00020
71	Propanoate metabolism	110	ko00640
72	Phenylalanine, tyrosine and tryptophan biosynthesis	107	ko00400
73	alpha-Linolenic acid metabolism	106	ko00592
74	Fatty acid metabolism	104	ko00071
75	Steroid biosynthesis	103	ko00100
76	beta-Alanine metabolism	98	ko00410
77	Sphingolipid metabolism	97	ko00600
78	Photosynthesis	93	ko00195
79	Natural killer cell mediated cytotoxicity	92	ko04650
80	Flavone and flavonol biosynthesis	91	ko00944
81	Ubiquinone and other terpenoid-quinone biosynthesis	89	ko00130
82	Glycosaminoglycan degradation	80	ko00531
83	Tryptophan metabolism	79	ko00380
84	Cutin, suberine and wax biosynthesis	75	ko00073
85	Fatty acid biosynthesis	74	ko00061

86	Pantothenate and CoA biosynthesis	74	ko00770
87	Diterpenoid biosynthesis	74	ko00904
88	Sulfur metabolism	68	ko00920
89	Valine, leucine and isoleucine biosynthesis	63	ko00290
90	Biosynthesis of unsaturated fatty acids	61	ko01040
91	Butanoate metabolism	60	ko00650
92	Lysine degradation	59	ko00310
93	Linoleic acid metabolism	55	ko00591
94	Tropane, piperidine and pyridine alkaloid biosynthesis	52	ko00960
95	Selenocompound metabolism	51	ko00450
96	Glycosphingolipid biosynthesis - ganglio series	48	ko00604
97	Isoquinoline alkaloid biosynthesis	47	ko00950
98	Non-homologous end-joining	47	ko03450
99	Isoflavonoid biosynthesis	46	ko00943
100	Vitamin B6 metabolism	46	ko00750
101	Circadian rhythm - mammal	45	ko04710
102	One carbon pool by folate	44	ko00670
103	Benzoxazinoid biosynthesis	43	ko00402
104	Riboflavin metabolism	41	ko00740
105	Folate biosynthesis	41	ko00790
106	Histidine metabolism	40	ko00340
107	Nicotinate and nicotinamide metabolism	39	ko00760
108	Lysine biosynthesis	37	ko00300
109	Other types of O-glycan biosynthesis	33	ko00514
110	Sulfur relay system	32	ko04122
111	Sesquiterpenoid and triterpenoid biosynthesis	32	ko00909
112	Arachidonic acid metabolism	31	ko00590
113	Photosynthesis - antenna proteins	30	ko00196
114	Brassinosteroid biosynthesis	30	ko00905
115	Fatty acid elongation	29	ko00062
116	Thiamine metabolism	27	ko00730
117	Taurine and hypotaurine metabolism	26	ko00430
118	Glucosinolate biosynthesis	24	ko00966
119	Glycosphingolipid biosynthesis - globo series	20	ko00603
120	Monoterpenoid biosynthesis	19	ko00902
121	C5-Branched dibasic acid metabolism	17	ko00660
122	Indole alkaloid biosynthesis	17	ko00901
123	Synthesis and degradation of ketone bodies	15	ko00072
124	Lipoic acid metabolism	12	ko00785
125	Anthocyanin biosynthesis	7	ko00942
126	Caffeine metabolism	6	ko00232
127	Biotin metabolism	5	ko00780
128	Betalain biosynthesis	1	ko00965

Supplementary Table S2. The enriched GO terms (Bonferroni-correction P ≤ 0.05) for DEGs in the comparisons of Control-A vs. COR-A and Control-B vs. COR-B

Comparison	Ontology	Description	Cluster frequency	Genome frequency of use	Corrected P-value
Control-A vs. COR-A	molecular_function	ADP binding	76/5741	240/28787	0.0141
	molecular_function	methionine adenosyltransferase activity	14/5741	23/28787	0.0260
	molecular_function	oxidoreductase activity, acting on paired donors, with incorporation or reduction of molecular oxygen	152/5741	564/28787	0.0391
	molecular_function	dioxygenase activity	80/5741	264/28787	0.0491
	biological_process	phenylpropanoid metabolic process	113/5668	378/28787	0.0018
	biological_process	S-adenosylmethionine biosynthetic process	14/5668	23/28787	0.0395
	biological_process	cellular modified amino acid metabolic process	70/5668	224/28787	0.0440
	biological_process	phenylpropanoid biosynthetic process	83/5668	277/28787	0.0452
Control-B vs. COR-B	molecular_function	catalytic activity	4031/6162	18025/28787	0.0002
	molecular_function	oxidoreductase activity, acting on paired donors, with incorporation or reduction of molecular oxygen	164/6162	564/28787	0.0125
	molecular_function	drug transporter activity	63/6162	179/28787	0.0200
	molecular_function	transferase activity, transferring phosphorus-containing groups	1013/6162	4251/28787	0.0267
	molecular_function	indole-3-acetic acid amido synthetase activity	9/6162	11/28787	0.0458

Supplementary Table S3. KEGG pathways of DEGs in the comparison of Control-A vs. COR-A

#	Pathway	Control-A-vs-COR-A DEGs Number (4820)	All-Unigene Number (25363)	P-value	Q-value	Pathway ID
1	Metabolic pathways	1073	5225	0.000882962	1.23E-02	ko01100
2	Biosynthesis of secondary metabolites	546	2386	3.81E-07	2.38E-05	ko01110
3	Plant-pathogen interaction	350	1592	0.001141237	1.30E-02	ko04626
4	Plant hormone signal transduction	304	1517	0.1524068	5.15E-01	ko04075
5	Spliceosome	178	1000	0.84895	9.99E-01	ko03040
6	RNA transport	150	960	0.997606	9.99E-01	ko03013
7	Purine metabolism	137	689	0.2896981	7.99E-01	ko00230
8	Protein processing in endoplasmic reticulum	134	713	0.573156	9.99E-01	ko04141
9	Pyrimidine metabolism	120	621	0.4349945	9.37E-01	ko00240
10	Phenylpropanoid biosynthesis	118	348	2.52E-11	3.15E-09	ko00940
11	Starch and sucrose metabolism	111	580	0.4839863	9.99E-01	ko00500
12	RNA degradation	101	502	0.2764591	7.99E-01	ko03018
13	Glycerophospholipid metabolism	95	528	0.741637	9.99E-01	ko00564
14	Endocytosis	90	629	0.9992888	9.99E-01	ko04144
15	RNA polymerase	89	403	0.065605	3.28E-01	ko03020
16	Ubiquitin mediated proteolysis	88	533	0.9400695	9.99E-01	ko04120
17	mRNA surveillance pathway	85	528	0.9642276	9.99E-01	ko03015
18	Ribosome biogenesis in eukaryotes	81	476	0.8809068	9.99E-01	ko03008
19	Ribosome	81	530	0.9896547	9.99E-01	ko03010
20	Cysteine and methionine metabolism	64	216	0.000103723	2.59E-03	ko00270
21	Oxidative phosphorylation	63	350	0.7057336	9.99E-01	ko00190
22	Ether lipid metabolism	63	387	0.9275515	9.99E-01	ko00565

23	ABC transporters	60	255	0.04090463	2.32E-01	ko02010
24	Stilbenoid, diarylheptanoid and gingerol biosynthesis	57	163	1.06E-06	4.42E-05	ko00945
25	Phagosome	57	271	0.216398	6.60E-01	ko04145
26	Amino sugar and nucleotide sugar metabolism	52	281	0.6088823	9.99E-01	ko00520
27	Flavonoid biosynthesis	51	184	0.002479101	2.38E-02	ko00941
28	Phenylalanine metabolism	50	166	0.000365963	6.55E-03	ko00360
29	Homologous recombination	50	221	0.1000088	4.46E-01	ko03440
30	Circadian rhythm - plant	44	247	0.7085308	9.99E-01	ko04712
31	Glycolysis / Gluconeogenesis	44	279	0.9306674	9.99E-01	ko00010
32	Terpenoid backbone biosynthesis	43	158	0.007265197	6.05E-02	ko00900
33	Peroxisome	42	227	0.603506	9.99E-01	ko04146
34	Arginine and proline metabolism	41	181	0.1240934	5.00E-01	ko00330
35	Pentose and glucuronate interconversions	40	226	0.7176484	9.99E-01	ko00040
36	Cyanoamino acid metabolism	39	152	0.02618221	1.56E-01	ko00460
37	Glycine, serine and threonine metabolism	38	165	0.1122802	4.74E-01	ko00260
38	Galactose metabolism	38	171	0.1635778	5.38E-01	ko00052
39	Glycerolipid metabolism	36	133	0.01432641	1.05E-01	ko00561
40	alpha-Linolenic acid metabolism	35	106	0.000419012	6.55E-03	ko00592
41	Phosphatidylinositol signaling system	35	188	0.5834843	9.99E-01	ko04070
42	Limonene and pinene degradation	34	125	0.0157484	1.09E-01	ko00903
43	Glutathione metabolism	32	154	0.3168332	7.99E-01	ko00480
44	Pyruvate metabolism	32	205	0.9113901	9.99E-01	ko00620
45	Valine, leucine and isoleucine degradation	31	122	0.04897543	2.55E-01	ko00280
46	Flavone and flavonol biosynthesis	30	91	0.00107359	1.30E-02	ko00944
47	Ascorbate and aldarate metabolism	30	127	0.1136604	4.74E-01	ko00053
48	Diterpenoid biosynthesis	29	74	4.29E-05	1.34E-03	ko00904

49	Basal transcription factors	28	167	0.7974284	9.99E-01	ko03022
50	Aminoacyl-tRNA biosynthesis	28	194	0.9611081	9.99E-01	ko00970
51	Cutin, suberine and wax biosynthesis	27	75	0.000401893	6.55E-03	ko00073
52	Glyoxylate and dicarboxylate metabolism	27	150	0.6556928	9.99E-01	ko00630
53	DNA replication	27	158	0.7602261	9.99E-01	ko03030
54	Inositol phosphate metabolism	27	175	0.9070259	9.99E-01	ko00562
55	Carotenoid biosynthesis	26	122	0.2904115	7.99E-01	ko00906
56	Mismatch repair	25	124	0.4063977	8.97E-01	ko03430
57	Zeatin biosynthesis	25	128	0.4750302	9.99E-01	ko00908
58	Glycosylphosphatidylinositol(GPI)-anchor biosynthesis	25	140	0.6689049	9.99E-01	ko00563
59	Nucleotide excision repair	25	208	0.9974777	9.99E-01	ko03420
60	Fructose and mannose metabolism	24	138	0.7184252	9.99E-01	ko00051
61	Base excision repair	24	160	0.9220536	9.99E-01	ko03410
62	Propanoate metabolism	23	110	0.3410741	8.20E-01	ko00640
63	Regulation of autophagy	23	147	0.8758208	9.99E-01	ko04140
64	Fatty acid metabolism	22	104	0.324455	7.99E-01	ko00071
65	Alanine, aspartate and glutamate metabolism	22	121	0.627713	9.99E-01	ko00250
66	Other glycan degradation	22	130	0.7602255	9.99E-01	ko00511
67	beta-Alanine metabolism	21	98	0.3071508	7.99E-01	ko00410
68	Tyrosine metabolism	21	113	0.583355	9.99E-01	ko00350
69	Sphingolipid metabolism	20	97	0.3817864	8.97E-01	ko00600
70	Proteasome	20	122	0.801232	9.99E-01	ko03050
71	Carbon fixation in photosynthetic organisms	20	155	0.9832506	9.99E-01	ko00710
72	Pantothenate and CoA biosynthesis	19	74	0.09701414	4.46E-01	ko00770
73	Steroid biosynthesis	19	103	0.5971057	9.99E-01	ko00100

74	Phenylalanine, tyrosine and tryptophan biosynthesis	19	107	0.6672057	9.99E-01	ko00400
75	Protein export	19	131	0.9273271	9.99E-01	ko03060
76	Valine, leucine and isoleucine biosynthesis	18	63	0.04265125	2.32E-01	ko00290
77	Nitrogen metabolism	18	126	0.9331007	9.99E-01	ko00910
78	Benzoxazinoid biosynthesis	17	43	0.001418501	1.48E-02	ko00402
79	Tryptophan metabolism	17	79	0.3260569	7.99E-01	ko00380
80	Pentose phosphate pathway	17	116	0.9093194	9.99E-01	ko00030
81	Fatty acid biosynthesis	16	74	0.3259911	7.99E-01	ko00061
82	Photosynthesis	16	93	0.7111494	9.99E-01	ko00195
83	SNARE interactions in vesicular transport	16	125	0.9750337	9.99E-01	ko04130
84	Isoflavonoid biosynthesis	15	46	0.01971932	1.23E-01	ko00943
85	Biosynthesis of unsaturated fatty acids	15	61	0.1698998	5.45E-01	ko01040
86	Sulfur metabolism	15	68	0.3043078	7.99E-01	ko00920
87	Ubiquinone and other terpenoid-quinone biosynthesis	15	89	0.7378504	9.99E-01	ko00130
88	N-Glycan biosynthesis	15	118	0.9736732	9.99E-01	ko00510
89	Linoleic acid metabolism	14	55	0.1474527	5.15E-01	ko00591
90	Butanoate metabolism	14	60	0.2392586	7.12E-01	ko00650
91	Citrate cycle (TCA cycle)	14	111	0.9722815	9.99E-01	ko00020
92	Porphyrin and chlorophyll metabolism	14	120	0.9887954	9.99E-01	ko00860
93	Vitamin B6 metabolism	12	46	0.1498338	5.15E-01	ko00750
94	Folate biosynthesis	11	41	0.1410221	5.15E-01	ko00790
95	Glycosaminoglycan degradation	11	80	0.91495	9.99E-01	ko00531
96	Natural killer cell mediated cytotoxicity	11	92	0.9741261	9.99E-01	ko04650
97	Glucosinolate biosynthesis	10	24	0.008758243	6.84E-02	ko00966
98	Nicotinate and nicotinamide metabolism	10	39	0.1933723	6.04E-01	ko00760
99	Isoquinoline alkaloid biosynthesis	10	47	0.4020969	8.97E-01	ko00950

100	Selenocompound metabolism	10	51	0.5124777	9.99E-01	ko00450
101	Lysine degradation	10	59	0.70697	9.99E-01	ko00310
102	Sesquiterpenoid and triterpenoid biosynthesis	9	32	0.1386616	5.15E-01	ko00909
103	Monoterpenoid biosynthesis	8	19	0.01729151	1.14E-01	ko00902
104	Histidine metabolism	8	40	0.499438	9.99E-01	ko00340
105	Tropane, piperidine and pyridine alkaloid biosynthesis	8	52	0.7973589	9.99E-01	ko00960
106	Riboflavin metabolism	7	41	0.6852274	9.99E-01	ko00740
107	Circadian rhythm - mammal	7	45	0.7776434	9.99E-01	ko04710
108	Glycosphingolipid biosynthesis - ganglio series	7	48	0.8325466	9.99E-01	ko00604
109	C5-Branched dibasic acid metabolism	6	17	0.08644505	4.16E-01	ko00660
110	Thiamine metabolism	6	27	0.4089434	8.97E-01	ko00730
111	Lysine biosynthesis	6	37	0.7306649	9.99E-01	ko00300
112	Anthocyanin biosynthesis	5	7	0.003685866	3.29E-02	ko00942
113	Synthesis and degradation of ketone bodies	5	15	0.1394634	5.15E-01	ko00072
114	Taurine and hypotaurine metabolism	5	26	0.5669747	9.99E-01	ko00430
115	Sulfur relay system	5	32	0.7543087	9.99E-01	ko04122
116	One carbon pool by folate	5	44	0.939548	9.99E-01	ko00670
117	Glycosphingolipid biosynthesis - globo series	4	20	0.5441189	9.99E-01	ko00603
118	Fatty acid elongation	4	29	0.8287942	9.99E-01	ko00062
119	Photosynthesis - antenna proteins	4	30	0.848666	9.99E-01	ko00196
120	Brassinosteroid biosynthesis	4	30	0.848666	9.99E-01	ko00905
121	Arachidonic acid metabolism	4	31	0.8665481	9.99E-01	ko00590
122	Non-homologous end-joining	4	47	0.9860538	9.99E-01	ko03450
123	Lipoic acid metabolism	3	12	0.4061889	8.97E-01	ko00785
124	Other types of O-glycan biosynthesis	3	33	0.9640365	9.99E-01	ko00514
125	Indole alkaloid biosynthesis	1	17	0.9722458	9.99E-01	ko00901

Supplementary Table S4. KEGG pathways of DEGs in the comparison of Control-B vs. COR-B

#	Pathway	Contror-B-vs-CORB Number (5283)	All-Unigene Number(25363)	P-value	Q-value	Pathway ID
1	Metabolic pathways	1108	5225	0.2316655	8.04E-01	ko01100
2	Biosynthesis of secondary metabolites	562	2386	0.00036932	1.40E-02	ko01110
3	Plant-pathogen interaction	328	1592	0.601303	1.00E+00	ko04626
4	Plant hormone signal transduction	294	1517	0.9295978	1.00E+00	ko04075
5	Spliceosome	196	1000	0.8454171	1.00E+00	ko03040
6	RNA transport	174	960	0.985089	1.00E+00	ko03013
7	Purine metabolism	141	689	0.6097804	1.00E+00	ko00230
8	Pyrimidine metabolism	138	621	0.2065728	7.38E-01	ko00240
9	Ribosome biogenesis in eukaryotes	116	476	0.03295687	3.55E-01	ko03008
10	Endocytosis	116	629	0.9402145	1.00E+00	ko04144
11	RNA degradation	114	502	0.1605308	7.09E-01	ko03018
12	Protein processing in endoplasmic reticulum	112	713	0.999822	1.00E+00	ko04141
13	Ubiquitin mediated proteolysis	109	533	0.6035128	1.00E+00	ko04120
14	Glycerophospholipid metabolism	108	528	0.6022402	1.00E+00	ko00564
15	Phenylpropanoid biosynthesis	105	348	2.27E-05	1.42E-03	ko00940
16	Starch and sucrose metabolism	105	580	0.9560409	1.00E+00	ko00500
17	RNA polymerase	92	403	0.1746262	7.09E-01	ko03020
18	Ribosome	83	530	0.9990626	1.00E+00	ko03010
19	mRNA surveillance pathway	78	528	0.9998703	1.00E+00	ko03015
20	Ether lipid metabolism	75	387	0.778137	1.00E+00	ko00565
21	Oxidative phosphorylation	63	350	0.9179483	1.00E+00	ko00190
22	Homologous recombination	62	221	0.006260867	1.30E-01	ko03440

23	Cysteine and methionine metabolism	59	216	0.01337298	1.86E-01	ko00270
24	ABC transporters	59	255	0.2007006	7.38E-01	ko02010
25	Peroxisome	55	227	0.1191208	7.09E-01	ko04146
26	Phagosome	52	271	0.7696482	1.00E+00	ko04145
27	Glycolysis / Gluconeogenesis	48	279	0.9448559	1.00E+00	ko00010
28	Amino sugar and nucleotide sugar metabolism	47	281	0.9649733	1.00E+00	ko00520
29	Stilbenoid, diarylheptanoid and gingerol biosynthesis	46	163	0.01493856	1.87E-01	ko00945
30	Circadian rhythm - plant	45	247	0.8638287	1.00E+00	ko04712
31	Cyanoamino acid metabolism	44	152	0.01079856	1.69E-01	ko00460
32	Glutathione metabolism	40	154	0.07245236	5.66E-01	ko00480
33	Base excision repair	40	160	0.1153973	7.09E-01	ko03410
34	Phenylalanine metabolism	40	166	0.1719797	7.09E-01	ko00360
35	Flavonoid biosynthesis	40	184	0.4088867	1.00E+00	ko00941
36	Nucleotide excision repair	40	208	0.7409674	1.00E+00	ko03420
37	DNA replication	39	158	0.1366737	7.09E-01	ko03030
38	Phosphatidylinositol signaling system	39	188	0.5405108	1.00E+00	ko04070
39	Basal transcription factors	38	167	0.2972835	9.07E-01	ko03022
40	Arginine and proline metabolism	38	181	0.5077128	1.00E+00	ko00330
41	Pentose and glucuronate interconversions	37	226	0.9621755	1.00E+00	ko00040
42	Limonene and pinene degradation	35	125	0.03408524	3.55E-01	ko00903
43	Glyoxylate and dicarboxylate metabolism	35	150	0.2522417	8.30E-01	ko00630
44	Terpenoid backbone biosynthesis	35	158	0.3709994	1.00E+00	ko00900
45	Glycosylphosphatidylinositol(GPI)-anchor biosynthesis	34	140	0.1815703	7.09E-01	ko00563
46	Aminoacyl-tRNA biosynthesis	34	194	0.8917961	1.00E+00	ko00970
47	alpha-Linolenic acid metabolism	33	106	0.008188357	1.46E-01	ko00592

48	Carotenoid biosynthesis	33	122	0.05981004	4.98E-01	ko00906
49	Inositol phosphate metabolism	33	175	0.7672603	1.00E+00	ko00562
50	Zeatin biosynthesis	32	128	0.1458931	7.09E-01	ko00908
51	Galactose metabolism	32	171	0.7796347	1.00E+00	ko00052
52	Nitrogen metabolism	31	126	0.1739124	7.09E-01	ko00910
53	Glycine, serine and threonine metabolism	31	165	0.7690429	1.00E+00	ko00260
54	Pyruvate metabolism	29	205	0.9946574	1.00E+00	ko00620
55	Mismatch repair	28	124	0.3487442	1.00E+00	ko03430
56	Other glycan degradation	28	130	0.4554266	1.00E+00	ko00511
57	Benzoxazinoid biosynthesis	27	43	2.87E-09	3.59E-07	ko00402
58	Alanine, aspartate and glutamate metabolism	27	121	0.3780948	1.00E+00	ko00250
59	Diterpenoid biosynthesis	26	74	0.003087113	7.72E-02	ko00904
60	Porphyrin and chlorophyll metabolism	26	120	0.4462661	1.00E+00	ko00860
61	SNARE interactions in vesicular transport	26	125	0.538774	1.00E+00	ko04130
62	Phenylalanine, tyrosine and tryptophan biosynthesis	25	107	0.293172	9.07E-01	ko00400
63	Glycerolipid metabolism	25	133	0.750029	1.00E+00	ko00561
64	Fatty acid metabolism	24	104	0.3216767	9.57E-01	ko00071
65	Valine, leucine and isoleucine degradation	24	122	0.6588147	1.00E+00	ko00280
66	Flavone and flavonol biosynthesis	23	91	0.1784412	7.09E-01	ko00944
67	Carbon fixation in photosynthetic organisms	23	155	0.9775128	1.00E+00	ko00710
68	Pentose phosphate pathway	22	116	0.7243392	1.00E+00	ko00030
69	Fructose and mannose metabolism	22	138	0.9399431	1.00E+00	ko00051
70	Ubiquinone and other terpenoid-quinone biosynthesis	21	89	0.2976157	9.07E-01	ko00130
71	Regulation of autophagy	21	147	0.9837768	1.00E+00	ko04140
72	Pantothenate and CoA biosynthesis	20	74	0.1223055	7.09E-01	ko00770

73	Sphingolipid metabolism	20	97	0.5606751	1.00E+00	ko00600
74	Tyrosine metabolism	20	113	0.8251161	1.00E+00	ko00350
75	Ascorbate and aldarate metabolism	19	127	0.9636023	1.00E+00	ko00053
76	Proteasome	18	122	0.9659078	1.00E+00	ko03050
77	Cutin, suberine and wax biosynthesis	17	75	0.3914262	1.00E+00	ko00073
78	Tryptophan metabolism	17	79	0.4842818	1.00E+00	ko00380
79	Propanoate metabolism	17	110	0.9386776	1.00E+00	ko00640
80	Natural killer cell mediated cytotoxicity	16	92	0.8263595	1.00E+00	ko04650
81	Citrate cycle (TCA cycle)	16	111	0.967539	1.00E+00	ko00020
82	Protein export	16	131	0.9964245	1.00E+00	ko03060
83	Isoflavonoid biosynthesis	15	46	0.04200294	4.04E-01	ko00943
84	beta-Alanine metabolism	15	98	0.9342224	1.00E+00	ko00410
85	Steroid biosynthesis	15	103	0.959458	1.00E+00	ko00100
86	Linoleic acid metabolism	14	55	0.2430468	8.21E-01	ko00591
87	Valine, leucine and isoleucine biosynthesis	14	63	0.4415942	1.00E+00	ko00290
88	Glycosaminoglycan degradation	14	80	0.8067947	1.00E+00	ko00531
89	Photosynthesis	14	93	0.938227	1.00E+00	ko00195
90	Sulfur metabolism	13	68	0.6825564	1.00E+00	ko00920
91	Riboflavin metabolism	12	41	0.1288351	7.09E-01	ko00740
92	Monoterpenoid biosynthesis	11	19	0.000447126	1.40E-02	ko00902
93	Histidine metabolism	11	40	0.1958906	7.38E-01	ko00340
94	Tropane, piperidine and pyridine alkaloid biosynthesis	11	52	0.5318175	1.00E+00	ko00960
95	Biosynthesis of unsaturated fatty acids	11	61	0.751704	1.00E+00	ko01040
96	N-Glycan biosynthesis	11	118	0.999758	1.00E+00	ko00510
97	Sesquiterpenoid and triterpenoid biosynthesis	10	32	0.1113856	7.09E-01	ko00909

98	One carbon pool by folate	10	44	0.4364166	1.00E+00	ko00670
99	Vitamin B6 metabolism	10	46	0.4975495	1.00E+00	ko00750
100	Glycosphingolipid biosynthesis - ganglio series	10	48	0.5567882	1.00E+00	ko00604
101	Selenocompound metabolism	10	51	0.6397835	1.00E+00	ko00450
102	Butanoate metabolism	10	60	0.8294923	1.00E+00	ko00650
103	Fatty acid biosynthesis	10	74	0.9609632	1.00E+00	ko00061
104	Brassinosteroid biosynthesis	9	30	0.1551643	7.09E-01	ko00905
105	Lysine biosynthesis	9	37	0.3608639	1.00E+00	ko00300
106	Nicotinate and nicotinamide metabolism	9	39	0.4261888	1.00E+00	ko00760
107	Folate biosynthesis	9	41	0.4910131	1.00E+00	ko00790
108	Isoquinoline alkaloid biosynthesis	8	47	0.7915161	1.00E+00	ko00950
109	Indole alkaloid biosynthesis	7	17	0.04594299	4.10E-01	ko00901
110	Lysine degradation	7	59	0.9750993	1.00E+00	ko00310
111	C5-Branched dibasic acid metabolism	6	17	0.1233464	7.09E-01	ko00660
112	Glucosinolate biosynthesis	6	24	0.3830423	1.00E+00	ko00966
113	Non-homologous end-joining	6	47	0.945986	1.00E+00	ko03450
114	Taurine and hypotaurine metabolism	5	26	0.6559512	1.00E+00	ko00430
115	Arachidonic acid metabolism	5	31	0.8037569	1.00E+00	ko00590
116	Thiamine metabolism	4	27	0.8438155	1.00E+00	ko00730
117	Fatty acid elongation	4	29	0.8820148	1.00E+00	ko00062
118	Photosynthesis - antenna proteins	4	30	0.8978657	1.00E+00	ko00196
119	Anthocyanin biosynthesis	3	7	0.1625673	7.09E-01	ko00942
120	Glycosphingolipid biosynthesis - globo series	3	20	0.8184006	1.00E+00	ko00603
121	Lipoic acid metabolism	2	12	0.7479781	1.00E+00	ko00785
122	Synthesis and degradation of ketone bodies	2	15	0.851232	1.00E+00	ko00072
123	Sulfur relay system	2	32	0.9946741	1.00E+00	ko04122

124	Other types of O-glycan biosynthesis	2	33	0.995667	1.00E+00	ko00514
125	Circadian rhythm - mammal	2	45	0.999653	1.00E+00	ko04710

Supplementary Table S5. Characterization of the DEGs in JA signalling pathway

Gene family	Unigene ID	Control-A vs. COR-A			Control-B vs. COR-B			Annotation (Alternative name)
		Fold Change	Up/ Down	FDR	Fold Change	Up/ Down	FDR	
JAZ homologues	CL5910.Contig1	10.07	Up	5.58E-05	11.23	Up	3.34E-10	Protein TIFY 10A OS=Arabidopsis thaliana (AtJAZ1)
	CL1826.Contig1	6.87	Up	1.50E-197				Protein TIFY 5A OS=Arabidopsis thaliana (AtJAZ8)
	Unigene36413	5.55	Up	5.58E-49	2.55	Up	1.26E-05	--
	CL4701.Contig2	4.25	Up	1.19E-68	2.49	Up	3.27E-13	Protein TIFY 10A OS=Arabidopsis thaliana (AtJAZ1)
	CL5181.Contig2	4.12	Up	2.62E-23				Protein TIFY 9 OS=Arabidopsis thaliana (AtJAZ10)
	CL5181.Contig1	4.05	Up	0				Protein TIFY 9 OS=Arabidopsis thaliana (AtJAZ10)
	Unigene8450	3.31	Up	0	-2.68	Down	1.52E-44	Protein TIFY 5A OS=Arabidopsis thaliana (AtJAZ8)
	CL1798.Contig2	2.58	Up	0				Protein TIFY 10A OS=Arabidopsis thaliana (AtJAZ1)
	CL1798.Contig1	2.55	Up	0				Protein TIFY 10B OS=Arabidopsis thaliana (AtJAZ2)
	Unigene11600	2.40	Up	1.39E-73				Protein TIFY 5A OS=Arabidopsis thaliana (AtJAZ8)
	CL4701.Contig1	2.11	Up	5.08E-133				Protein TIFY 10A OS=Arabidopsis thaliana (AtJAZ1)
	Unigene12451	2.11	Up	1.99E-19				Protein TIFY 10A OS=Arabidopsis thaliana (AtJAZ1)
	CL1798.Contig3	1.84	Up	0				Protein TIFY 10A OS=Arabidopsis thaliana (AtJAZ1)
	Unigene6174	1.75	Up	1.72E-21				--
	CL9834.Contig2	1.72	Up	2.46E-25				Protein TIFY 6B OS=Arabidopsis thaliana (AtJAZ3)
	CL9834.Contig1	1.49	Up	1.12E-07				Protein TIFY 6B OS=Arabidopsis thaliana (AtJAZ3)
	CL9834.Contig5	1.43	Up	8.32E-14	-4.82	Down	1.43E-07	Protein TIFY 6B OS=Arabidopsis thaliana (AtJAZ3)
	CL9834.Contig3	1.41	Up	1.50E-13				Protein TIFY 6B OS=Arabidopsis thaliana (AtJAZ3)
	CL2710.Contig1	1.23	Up	1.27E-86				Protein TIFY 6B OS=Arabidopsis thaliana (AtJAZ3)

	CL2967.Contig1	1.16	Up	0				Protein TIFY 3B OS=Arabidopsis thaliana (AtJAZ12)
	CL2710.Contig3	1.14	Up	2.61E-25				Protein TIFY 6B OS=Arabidopsis thaliana (AtJAZ3)
	CL2710.Contig2	1.13	Up	2.83E-99				Protein TIFY 6B OS=Arabidopsis thaliana (AtJAZ3)
	CL2710.Contig5	1.13	Up	1.50E-10				Protein TIFY 6B OS=Arabidopsis thaliana (AtJAZ3)
	CL9834.Contig4	1.04	Up	6.59E-06				Protein TIFY 6B OS=Arabidopsis thaliana (AtJAZ3)
	CL1965.Contig2	-1.97	Down	9.39E-12				Protein TIFY 8 OS=Arabidopsis thaliana
	Unigene12832				1.24	Up	5.40E-04	Protein TIFY 4B OS=Arabidopsis thaliana
MYC homologues	Unigene16394	11.20	Up	4.40E-15				Transcription factor bHLH79 OS=Arabidopsis thaliana
	CL4488.Contig2	4.18	Up	2.21E-24				Transcription factor bHLH63 OS=Arabidopsis thaliana
	CL3407.Contig2	3.11	Up	1.64E-21	-5.75	Down	8.53E-15	Transcription factor bHLH62 OS=Arabidopsis thaliana
	CL10929.Contig4	2.88	Up	3.43E-75				Transcription factor bHLH35 OS=Arabidopsis thaliana
	CL10929.Contig3	2.86	Up	1.77E-36				Transcription factor bHLH35 OS=Arabidopsis thaliana
	CL3854.Contig5	2.59	Up	3.62E-28				Transcription factor MYC2 OS=Arabidopsis thaliana
	Unigene8274	2.53	Up	1.58E-20				Transcription factor GLABRA 3 OS=Arabidopsis thaliana (AtMYC6)
	CL630.Contig2	2.15	Up	1.95E-258	-1.03	Down	5.70E-18	Transcription factor bHLH13 OS=Arabidopsis thaliana
	CL8303.Contig1	2.14	Up	3.14E-15				Transcription factor GLABRA 3 OS=Arabidopsis thaliana (AtMYC6)
	CL3854.Contig6	2.10	Up	4.31E-28				Transcription factor MYC2 OS=Arabidopsis thaliana
	CL630.Contig3	1.99	Up	9.02E-60				Transcription factor bHLH13 OS=Arabidopsis thaliana
	Unigene16392	1.81	Up	8.35E-08	1.62	Up	1.97E-04	Transcription factor bHLH79 OS=Arabidopsis thaliana
	CL3854.Contig4	1.66	Up	7.87E-32	1.09	Up	1.52E-12	Transcription factor MYC2 OS=Arabidopsis thaliana
	CL3574.Contig3	1.60	Up	1.08E-10				Transcription factor BIM1 OS=Arabidopsis thaliana (AtbHLH46, BES1-interacting Myc-like protein 1)
	CL3407.Contig1	1.56	Up	3.56E-56				Transcription factor bHLH78 OS=Arabidopsis thaliana
	CL630.Contig4	1.46	Up	1.38E-87				Transcription factor bHLH13 OS=Arabidopsis thaliana

CL3574.Contig4	1.51	Up	9.96E-07				Transcription factor BIM1 OS=Arabidopsis thaliana (AtbHLH46, BES1-interacting Myc-like protein 1)
CL9129.Contig1	1.45	Up	1.21E-10	1.05	Up	1.45E-06	Transcription factor TT8 OS=Arabidopsis thaliana (AtbHLH42)
CL3854.Contig3	1.39	Up	1.10E-291				Transcription factor MYC2 OS=Arabidopsis thaliana
CL6121.Contig2	1.41	Up	3.87E-33				Transcription factor bHLH104 OS=Arabidopsis thaliana
CL8303.Contig2	1.39	Up	1.40E-11				Transcription factor GLABRA 3 OS=Arabidopsis thaliana (AtMYC6)
CL4234.Contig3	1.33	Up	9.26E-16				Transcription factor GLABRA 3 OS=Arabidopsis thaliana (AtMYC6)
CL3574.Contig2	1.30	Up	1.10E-25				Transcription factor BIM1 OS=Arabidopsis thaliana (AtbHLH46, BES1-interacting Myc-like protein 1)
CL3854.Contig1	1.21	Up	1.65E-09				Transcription factor MYC2 OS=Arabidopsis thaliana
CL8472.Contig1	1.11	Up	3.97E-74				Transcription factor bHLH94 OS=Arabidopsis thaliana
CL11065.Contig1	-2.71	Down	1.58E-07	-2.72	Down	6.32E-06	Transcription factor bHLH57 OS=Arabidopsis thaliana
Unigene3315	-2.59	Down	2.24E-19				Transcription factor AIG1 OS=Arabidopsis thaliana (AtbHLH32)
CL7093.Contig2	-1.82	Down	3.12E-22				Transcription factor BEE 3 OS=Arabidopsis thaliana (AtbHLH50)
CL1585.Contig2	-1.79	Down	4.29E-19				Transcription factor bHLH93 OS=Arabidopsis thaliana
CL8756.Contig1	-1.77	Down	1.22E-29	-2.05	Down	1.83E-90	Transcription factor BEE 3 OS=Arabidopsis thaliana (AtbHLH50)
Unigene5897	-1.58	Down	1.43E-19				Transcription factor bHLH30 OS=Arabidopsis thaliana
CL8019.Contig1	1.15	Up	4.16E-07				--
CL3854.Contig2	2.52	Up	5.31E-04				Transcription factor MYC2 OS=Arabidopsis thaliana
CL7369.Contig1	1.73	Up	8.65E-15				Transcription factor UNE12 OS=Arabidopsis thaliana

				(AtbHLH59)
CL10929.Contig1	-9.69	Down	4.43E-06	Transcription factor bHLH35 OS=Arabidopsis thaliana
CL1605.Contig1	-9.45	Down	2.44E-06	Transcription factor bHLH79 OS=Arabidopsis thaliana
Unigene10892	-8.25	Down	8.88E-05	Transcription factor bHLH66 OS=Arabidopsis thaliana
CL8303.Contig5	-8.13	Down	2.94E-04	Transcription factor GLABRA 3 OS=Arabidopsis thaliana (AtMYC6)
CL9129.Contig2	-5.36	Down	3.91E-11	Transcription factor TT8 OS=Arabidopsis thaliana (AtbHLH42)
CL5337.Contig1	-3.26	Down	2.55E-27	Transcription factor ICE1 OS=Arabidopsis thaliana (AtbHLH116)
CL3407.Contig3	-1.79	Down	9.19E-06	Transcription factor bHLH78 OS=Arabidopsis thaliana
CL3139.Contig1	-1.47	Down	4.17E-35	Transcription factor bHLH106 OS=Arabidopsis thaliana
Unigene612	-1.39	Down	1.08E-20	Transcription factor bHLH96 OS=Arabidopsis thaliana
CL4488.Contig5	-1.28	Down	1.09E-13	Transcription factor bHLH63 OS=Arabidopsis thaliana
Unigene9129	-1.16	Down	3.25E-07	Transcription factor bHLH30 OS=Arabidopsis thaliana
CL9057.Contig2	-1.09	Down	3.35E-10	Transcription factor bHLH30 OS=Arabidopsis thaliana

Supplementary Table S6. Characterization of the DEGs in CLAVATA-MAPK-WOX signalling pathway

Gene family	Unigene ID	Control-A vs. COR-A			Control-B vs. COR-B			Annotation
		Fold Change	Up/ Down	FDR	Fold Change	Up/ Down	FDR	
CLAVATA homologues	Unigene19900				1.55	Up	4.66E-07	CLAVATA3/ESR (CLE)-related protein 1
	CL11164.Contig2				-2.21	Down	2.99E-20	CLAVATA3/ESR (CLE)-related protein 12
	CL6064.Contig2	-1.24	Down	2.60E-22				CLAVATA3/ESR (CLE)-related protein 25
	CL6064.Contig3	-3.34	Down	1.98E-41				CLAVATA3/ESR (CLE)-related protein 25
	CL8191.Contig2				-2.60	Down	7.28E-09	CLAVATA3/ESR (CLE)-related protein 46
	CL821.Contig2	-2.00	Down	5.44E-07	-12.13	Down	7.49E-24	CLAVATA3/ESR (CLE)-related protein TDIF
	CL5640.Contig4				-1.06	Down	4.19E-55	CLAVATA3/ESR (CLE)-related protein TDIF
	Unigene1369	2.58	Up	1.51E-28	2.49	Up	6.29E-17	Receptor protein kinase CLAVATA1
	Unigene27145	3.97	Up	9.59E-24	2.89	Up	6.74E-19	Receptor protein kinase CLAVATA1
	CL9218.Contig1	2.02	Up	6.12E-33	1.17	Up	1.36E-13	Receptor protein kinase CLAVATA1
	CL906.Contig1	-1.17	Down	6.15E-13				Receptor protein kinase CLAVATA1
MAPK cascades	CL2617.Contig3				11.02	Up	5.44E-09	Mitogen-activated protein kinase kinase kinase 1
	CL3286.Contig1	-1.42	Down	1.04E-06	1.05	Up	2.24E-06	Mitogen-activated protein kinase kinase kinase 1
	CL5004.Contig1	2.52	Up	3.91E-21				Mitogen-activated protein kinase kinase kinase 2
	Unigene27389				4.30	Up	4.89E-05	Mitogen-activated protein kinase kinase kinase 2
	CL2431.Contig1	1.86	Up	2.18E-50	-5.50	Down	3.81E-24	Mitogen-activated protein kinase kinase kinase 3
	CL2431.Contig2	2.34	Up	0				Mitogen-activated protein kinase kinase kinase 3
	CL2431.Contig3	1.94	Up	4.57E-253				Mitogen-activated protein kinase kinase kinase 3
	CL2958.Contig3				-11.90	Down	6.50E-08	Mitogen-activated protein kinase kinase kinase 3
	CL10672.Contig3				3.26	Up	1.81E-07	Mitogen-activated protein kinase kinase kinase 3

	CL7383.Contig1				-11.27	Down	8.92E-05	Mitogen-activated protein kinase kinase kinase
	Unigene35052				-11.74	Down	1.48E-05	Mitogen-activated protein kinase kinase kinase
	CL3831.Contig3				3.94	Up	3.84E-13	Mitogen-activated protein kinase homolog MMK2
	Unigene25401				2.09	Up	6.29E-17	Mitogen-activated protein kinase kinase
	Unigene21469	1.61	Up	2.01E-05	12.35	Up	1.66E-10	Mitogen-activated protein kinase 6
WOX homologues	Unigene5108	-2.81	Down	1.58E-12				WUSCHEL-related homeobox 5
	Unigene9698	-1.49	Down	3.31E-10	-1.13	Down	4.45E-04	WUSCHEL-related homeobox 5
	CL8639.Contig2				-1.29	Down	1.59E-06	WUSCHEL-related homeobox 13

Supplementary Table S7. The relative expression (Log 2 value) of the unigenes in CLAVATA-MAPK-WOX signalling pathway by qRT-PCR

Gene family	Unigene ID	Treatment time						
		1 h	2 h	4 h	8 h	1 d	2 d	3 d
CLAVATA homologues	Unigene19900	-1.9428	-1.7400	0.8064	2.3142	0.6867	-0.7693	2.8390
	CL11164.Contig2	1.0276	-1.5395	-0.7697	-2.0827	1.0771	-1.2380	-1.5046
	CL6064.Contig2	-0.6418	-0.1159	-0.0726	0.8435	-0.7823	-0.5162	-0.6754
	CL6064.Contig3	0.1834	0.1302	0.3045	0.8539	1.2201	-0.3466	-0.3672
	CL8191.Contig2	-0.1200	0.0239	-0.4518	-1.4884	0.1037	-0.1438	-0.1076
	CL821.Contig2	-0.0062	-0.2236	-0.2599	-0.2487	-0.2460	-0.0488	-0.2685
	CL5640.Contig4	-0.2761	-0.3666	-2.2156	-0.8475	-0.2017	-0.4335	0.0035
	Unigene1369	0.4661	2.2358	2.4706	4.3408	3.8620	-0.1202	1.3025
	Unigene27145	0.3296	2.5587	3.1309	4.3285	3.3206	0.0527	1.1419
	CL9218.Contig1	-0.2516	1.5990	1.1672	3.1765	0.5407	-0.1593	0.6889
	CL906.Contig1	-0.8415	0.0410	-0.6441	-0.7345	-0.6686	-1.0416	0.0378
MAPK cacades	CL2617.Contig3	-0.1681	0.2312	-0.2347	0.2087	-0.3265	-0.0965	-0.0655
	CL3286.Contig1	-0.1049	-1.0750	-1.2308	-1.1484	-0.5597	0.7194	-0.0856
	CL5004.Contig1	-0.8264	-0.0669	-0.1406	0.0016	-0.6918	-0.6077	-0.5112
	Unigene27389	-0.1198	0.7180	0.3989	1.4426	0.2651	0.4203	0.0621
	CL2431.Contig1	0.5380	2.5955	1.3838	3.5683	1.2488	-0.3543	0.9680
	CL2431.Contig2	0.8541	2.3778	1.0655	2.5660	1.6827	-0.6105	0.9989
	CL2431.Contig3	0.7342	2.7270	2.4879	3.4862	1.3923	-0.3715	1.0283
	CL2958.Contig3	-0.3223	0.1238	-0.0311	-0.4957	-1.7572	-0.0418	0.0804

	CL10672.Contig3	-0.2327	0.0227	-0.3976	-0.2131	-2.3907	-1.0775	-0.1093
	CL7383.Contig1	-0.0162	0.7823	-0.1114	0.4728	0.6224	-0.5895	-0.4901
	Unigene35052	-0.6487	-1.4616	-0.0071	0.1533	-0.7826	-0.5698	-0.3188
	CL3831.Contig3	0.0271	0.1697	-0.2712	-0.9663	0.0996	0.6740	-0.0964
	Unigene25401	-0.3521	0.3649	-0.0330	0.2036	-0.2086	0.0541	-0.0914
	Unigene21469	0.2407	-0.6176	-0.2341	-0.5305	-0.7254	0.3274	-0.1620
WOX homologues	Unigene5108	-0.4497	0.3109	-0.1382	-0.4388	-0.3181	0.0099	-0.3219
	Unigene9698	0.1252	0.4065	0.1926	0.3750	0.6659	0.5865	-0.0727
	CL8639.Contig2	-0.7851	-0.4260	0.0901	-0.9067	-0.1461	0.5339	-0.0416

Supplementary Table S8. Characterization of the DEGs in Ca^{2+} signal transduction

Gene family	Unigene ID	Control-A vs. COR-A			Control-B vs. COR-B			Annotation
		Fold Change	Up/ Down	FDR	Fold Change	Up/ Down	FDR	
CAMTA homologues	CL2775.Contig1	1.39	Up	8.30E-08	-11.00	Down	2.33E-11	Calmodulin-binding transcription activator 2
	Unigene7743	-1.23	Down	3.00E-10	-1.33	Down	2.65E-07	Calmodulin-binding transcription activator 3
	CL6264.Contig1	-1.86	Down	5.37E-05				Calmodulin-binding transcription activator 4
	Unigene17796				1.56	Up	5.48E-12	Calmodulin-binding transcription activator (camta)
	CL2775.Contig2				1.06	Up	2.40E-04	Calmodulin-binding transcription activator 1
CABP homologues	Unigene33354	4.79	Up	3.47E-07	-11.54	Down	2.69E-05	Calmodulin binding protein
	Unigene21291	2.09	Up	1.18E-09	-1.97	Down	1.21E-06	Calmodulin binding protein
	Unigene2189	1.92	Up	6.42E-08				Calmodulin binding protein
	CL32.Contig1	1.91	Up	1.09E-10				Calmodulin binding protein
	Unigene17199	1.54	Up	1.28E-11				Calmodulin binding protein
	Unigene25748	1.49	Up	3.82E-06	-1.69	Down	1.30E-05	Calmodulin binding protein
	Unigene1602	1.44	Up	1.43E-12				Calmodulin binding protein
	Unigene7412	1.38	Up	5.52E-04	-12.54	Down	1.07E-12	Calmodulin binding protein
	CL7268.Contig3	1.35	Up	4.21E-08	-1.61	Down	4.80E-10	Calmodulin binding protein
	CL11435.Contig1	1.01	Up	6.21E-06				Calmodulin binding protein
	Unigene23007	1.01	Up	8.54E-05				Calmodulin binding protein
	CL7904.Contig1	-1.11	Down	2.95E-05				Calmodulin binding protein
	CL6311.Contig2	-1.13	Down	1.36E-04				Calmodulin binding protein
	CL2659.Contig5	-2.26	Down	6.64E-05				Calmodulin binding protein
	Unigene17248	-6.15	Down	3.55E-19	1.83	Up	3.64E-15	Calmodulin binding protein

	CL7496.Contig2	-11.21	Down	3.06E-04				Calmodulin binding protein
	Unigene16974	-11.83	Down	6.85E-08	1.55	Up	3.95E-11	Calmodulin binding protein
	Unigene2396	-12.04	Down	3.54E-08	-12.20	Down	1.93E-08	Calmodulin binding protein
	CL2659.Contig2				1.08	Up	6.08E-05	Calmodulin binding protein
	Unigene19761				-1.30	Down	7.60E-07	Calmodulin binding protein
	CL9329.Contig1				-1.57	Down	3.14E-17	Calmodulin binding protein
	Unigene23223				-1.63	Down	0.00031132	Calmodulin binding protein
	CL6779.Contig1				-1.70	Down	2.37E-07	Calmodulin binding protein
	Unigene24592				-2.02	Down	3.86E-60	Calmodulin binding protein
	CL10807.Contig2				-2.09	Down	1.85E-11	Calmodulin binding protein
	Unigene12739				-3.64	Down	2.52E-08	Calmodulin binding protein
	Unigene23943				-11.42	Down	5.72E-09	Calmodulin binding protein
	Unigene34740				-12.04	Down	6.53E-08	Calmodulin binding protein
CDPK homologues	CL553.Contig3	2.91	Up	2.18E-10	-9.89	Down	4.89E-05	Calcium-dependent protein kinase (CDPK2)
	CL11211.Contig1	2.54	Up	3.49E-27				Calcium-dependent protein kinase (CDPK2)
	Unigene1251	1.98	Up	1.06E-07	1.57	Up	3.13E-07	Calcium-dependent protein kinase 6 (CDPK6)
	CL6154.Contig2	1.66	Up	3.90E-21	-1.25	Down	2.15E-08	Calcium-dependent protein kinase (CDPK1)
	CL553.Contig1	1.21	Up	2.55E-07				Calcium-dependent protein kinase (CDPK2)
	CL553.Contig2	1.15	Up	3.71E-09				Calcium-dependent protein kinase (CDPK2)
	Unigene11602	1.01	Up	1.97E-08				Calcium-dependent protein kinase 4
	Unigene14446	-1.47	Down	2.90E-04	1.50	Up	1.39E-09	Calcium-dependent protein kinase 29
	Unigene37120				13.46	Up	1.90E-24	Calcium-dependent protein kinase 1 (CDPK1)
	Unigene37409				10.02	Up	2.12E-05	Calcium-dependent protein kinase 2
	Unigene38196				8.06	Up	0	Calcium-dependent protein kinase 1 (CDPK1)
	CL11150.Contig2				3.76	Up	0	Calcium-dependent protein kinase 1 (CDPK1)
	Unigene8289				2.72	Up	8.96E-04	Calcium-dependent protein kinase 1

CL915.Contig4	2.04	Up	1.31E-25	Calcium-dependent protein kinase 9
CL10487.Contig1	1.99	Up	1.06E-22	Calcium-dependent protein kinase
CL11150.Contig4	1.66	Up	9.16E-07	Calcium-dependent protein kinase 1 (CDPK1)
CL1288.Contig4	1.40	Up	8.44E-15	Calcium-dependent protein kinase 4
CL6815.Contig6	1.10	Up	1.25E-05	Calcium-dependent protein kinase SK5
Unigene2737	1.00	Up	4.78E-06	Calcium-dependent protein kinase 9
CL6815.Contig5	-1.11	Down	6.30E-16	Calcium-dependent protein kinase SK5
Unigene1447	-1.27	Down	2.85E-10	Calcium-dependent protein kinase 21
CL5907.Contig1	-5.78	Down	4.65E-15	Calcium-dependent protein kinase 1
Unigene4688	-11.38	Down	3.13E-09	Calcium-dependent protein kinase 20
Unigene33474	-11.59	Down	1.48E-05	Calcium-dependent protein kinase 1 (CDPK1)

Supplementary Table S9. Primers used in this paper

Unigene ID	Real-time PCR primers: forward (5'-3')	Real-time PCR primers: reverse(5'-3')	Description
Unigene3058	GACACAGGACGTGCAGAGTT	TGGGTAAAGTTGGACAAAGTTTCTC	For validation of digital gene expression in Control-A vs. COR-A
Unigene6485	CGCTGAAGTCTGTGAGTCCAT	CTCCTCTTGACCGTGACCTTC	
CL7132.Contig2	TGGAGAAGTGGTTGGATTATTGGA	CCTACACGCTGACATGAACATG	
Unigene22560	CATTGGAGGAAGAGGAGGAAGG	AACTCAAGACCAGAGCCACTTAA	
CL9659.Contig1	TGATCCACTCAAGTATGATCCAGAA	CTCGCCAGCAACATCGTAGT	
CL1798.Contig1	CTTCGCAGTAACACGATTGCTAATCA	GCAGGATTCAACATCCGCCACAA	
Unigene11600	GCGGAGATGGCAAACTTGGAGAA	CACCTGTATTGGAGGAGAAGGAGACT	
CL4023.Contig3	ACCAGAGACTGAGCATCCAAC	GACAATTCCACAACTGCCTTAGAA	
CL1309.Contig2	CAAGCAGCATTTACGACAGAGAA	GCATACAGCACCTTCACACTTC	
CL6121.Contig2	TAATCACAGTGGTGGCGTGGATATTG	GGTTTGTCAGTTCTGGCAGGTCTC	
CL2967.Contig1	CAGAGTGTGCTGGGAATGATGATGT	ATGGCGATTAGGCAACTATGTGAACA	
CL8019.Contig1	CGTGACCTGTTGGAGCCATTCTATT	CCACCCTCCTCCTCTTCCTTCTTT	
CL2710.Contig2	GAGAGAGCTGATGTAGGAGTTCCTTT	CCACAATGCGACGGAGAGTTGA	
CL1304.Contig1	AGAGGAGGACATGAGGAGACAA	TAGAACGCACTACCGAACATAGG	
CL7093.Contig2	TCGTTCCTGGATGCCACAAG	AACTCAGCTCTCCATATCCTTCTC	
CL1965.Contig2	CCAAGGAGAACCAAGCACTGAAGATA	GGCCATGTCTACATCAGATCAAGAAT	
Unigene12439	GGTGGAAACAATGGAGCAAATGA	AGGTCTGTTATCTCTCACATCAGTA	
Unigene8372	GCGGAGAGTTCAGGCATGT	GTTCACTTACACGAGGACTATACG	
CL1582.Contig1	GGCTAAGGTGGAGGCAACAA	CACTCAAGTTCAATCTCAGACATCT	
Unigene7614	GTTCCATGTTCGTTGTTGTTCCT	TCTTAGCAGCACCTTCCTTGAC	
Unigene6485	CGCTGAAGTCTGTGAGTCCAT	CTCCTCTTGACCGTGACCTTC	For validation of digital gene expression in Control-B vs. COR-B
CL4808.Contig1	AGGTAGCACTGGCATGATTGG	GGTGAGATTCGGAGTTGTTGGT	

Unigene14030	AGCAGCAGCAGCAGCAAT	TGTGAGGTTGGCGTTAGTATTAGTA	
CL9659.Contig1	TGATCCACTCAAGTATGATCCAGAA	CTCGCCAGCAACATCGTAGT	
CL11184.Contig2	CTACCGAGCCAAGTGCCATTA	ATAGAACAACACTAGAAGCCAGAGA	
Unigene3905	TTGCTGTGTTCATGGTTCATCTG	ATGCCTTGTCCTGGTTGTAGAT	
CL1790.Contig3	GGAGGCTTGGTTGGTGGAATACA	ACCTGCTCGCTGTGTCCTTGA	
CL3741.Contig10	CAGGCAGCATTGAAGTGGAGTA	TTGATGGAGACGATGAGTCAGAC	
CL7132.Contig2	TGGAGAAGTGGTTGGATTATTGGA	CCTACACGCTGACATGAACATG	
CL271.Contig2	GCCTCTTGTTCACTGCTTACTTC	AAGGACCCGAAACCCAGAAAG	
Unigene14220	CCAGGAGAGGACTTCAGCTCATTCA	AGCAAGCGGTTGACAGAGATTGAC	
CL1582.Contig1	GGCTAAGGTGGAGGCAACAA	CACTCAAGTTCAATCTCAGACATCT	
Unigene5791	GCAGCAGTTGAAGTTGGAATGG	AGCAGAAATGGAGAAGCAGGAA	
CL4489.Contig8	GAGAAGAGGAGGAAGAGGAGAATG	CCTTATCAGGAAACAGAACTCACAT	
Unigene3058	GACACAGGACGTGCAGAGTT	TGGGTAAAGTTGGACAAAGTTTCTC	
CL7024.Contig3	GGCTGTGAGTTGGCGTGAT	CCTTCGCACCACACATTATTTGA	
CL7093.Contig2	TCGTTCCTGGATGCCACAAG	AACTCAGCTCTCCATATCCTTCTC	
Unigene8601	GCCTTGAAGAACTGCGTAAGAAT	CACCAAGCAAGACAACAGACATAT	
Unigene5855	TCTTAGGAACAAGGAGGAAGGTTAT	CCTCTCTCAATCTTCTTTCTGCTTT	
CL3407.Contig2	CCAACATTCTGTGAGGATGATCTC	TATCTGGAGTCTGGACCTTCAATC	
CL1798.Contig1	CTTCGCAGTAACACGATTGCTAATCA	GCAGGATTCAACATCCGCCACAA	For the qRT-PCR analysis of *t*he homologous unigenes matched to *JAZ*
CL1798.Contig3	GGGTTGAATCCTGCTGTTTCCTTATG	TCGTGTTTCATGCGAAATTCCTGTA	
CL2710.Contig2	GAGAGAGCTGATGTAGGAGTTCCTTT	CCACAATGCGACGGAGAGTTGA	
CL2710.Contig5	CACCAAGGAAGGAGAGTAACCACAAT	AAACACCCTACACCAGCTCTCTC	
CL2967.Contig1	CAGAGTGTGCTGGGAATGATGATGT	ATGGCGATTAGGCAACTATGTGAACA	
CL4701.Contig1/2	GACCAAGTTCGCCATATCGCTCAG	ACAGCCACCATCCATTGTTTCTTCAT	
CL5181.Contig2	GGGTAAACGTGGAAATGGAGTCTGT	AAGTCTGTCATTGCCTCTGGTTCTG	
CL9834.Contig2	CATCTCCATGCTCAGTCCTTCCAAG	CAGGTGCTGCTGAACTAACTGTCTT	

CL9834.Contig3	AAGGATTCGGTTCCAATGAGAGGTTC	ACATGGCTTCTGGTTAGCAGTTGAA	
CL9834.Contig4	TCCGCTGAATATGATGGTGTGAGTT	TCTCTTCCTTCATATTTGTGGAGTGC	
Unigene6174	TGCCACTGAATGTGATGATGTGAGTT	AGCTGTAGAGTGCATGGTATTGGATT	
Unigene11600	GCGGAGATGGCAAACTTGGAGAA	CACCTGTATTGGAGGAGAAGGAGACT	
Unigene12451	TGCCTTTGATGCTACCCTATCTTTCC	CTACGACTGCTTCTACTTCTGCCATG	
CL630.Contig2	CGCATTCGTGGTGAAGAACCCTTT	ATTTGCTGCCCTACTACTTGGTCAAT	For the qRT-PCR analysis of the homologous unigenes matched to *MYC*
CL3574.Contig2	GGAATCCCATCAAGCCTCAAAGAAG	GCATAAACTCCCATGACGACACAG	
CL3854.Contig1/4/5	TTAATGGAGGAGCTGAACTGAGGAGT	GATAGCACAAAGCCCATCAGAGGTT	
CL3854.Contig6	TCAGGAATTGCGTTTAGCACTTGGA	GCTCCTCAGTTCAATCAGCTCATCC	
CL4234.Contig3	GAGAGGCAGAGATGGATTATGGCATAT	TGAACCGAGTGAGAATCTAACAGCAG	
CL6121.Contig2	TAATCACAGTGGTGGCGTGGATATTG	GGTTTGTCAGTTCTGGCAGGTCTC	
CL8019.Contig1	CGTGACCTGTTGGAGCCATTCTATT	CCACCCTCCTCCTCTTCCTTCTTT	
CL8303.Contig1	ATTCTCACAGCGAGCCTAAAGTCTAA	CAACTCTTAATTACTGCCAACCTTCCA	
CL10929.Contig3	CTGCTTCTTCGGCGGCTTCTAAG	GCTCTTGCTCAAAGTCATAACCAGGA	
Unigene5897	TGATCCTTCCTTCTCATCTTCATCAGG	CGCCAAATTAACAAGCATACGGACATA	
Unigene10892	GGGTCTTTATATTCTGGCAGTGTTTCA	GTTGGGTGGTGCGGTTGGTA	
Unigene19900	TCGTCCAATCCACCACTTCTCACA	ACCTCCAATCATCTCTTGCCTCCTAA	For the qRT-PCR analysis of DEGs in CLAVATA-MAPK-WOX signalling pathway
CL11164.Contig2	AAGAAGCACCACCATCAACACCAT	CGACACCATAACGAGGATCAATCTCAT	
CL6064.Contig2	CACTCTATCTCCTCACCTCACCCTTT	GGCACAGAACCGCTACTAATAACCAT	
CL6064.Contig3	CCATAAGTACAAGGCATCTTCCTCCAT	TTGGTTGCTTCAGTTGGCAGAGTT	
CL8191.Contig2	AGTAGGCAGATTCTCTTCCGTCTTCT	CCTTGTGCGATCTTGAGCTTGGT	
CL821.Contig2	ACCACCTTATGCTTCTTCTTCTGTTG	CCCTCTTTGCTTACTTGTTGGATTCA	
CL5640.Contig4	GCTCGCACGATTCTTCTTCAACAA	TTCACCGTTACAACTCTAGCATCTTCA	
Unigene1369	ATATAGGTTGGCGGACAGCATACAC	GCTTCCAGCAGTCATGGCTACAA	
Unigene27145	ACCAATTCCTGAGGAGCTAGGTTACT	TGGTTAATGCAGGCATGTTGAAGATTC	

CL9218.Contig1	GTCACTATGTCCACTCCATCTCCAA	ATTCGCTTACACGCTCAAGGTAGA	
CL906.Contig1	GAGAACTGCCTTATGAGATGTCTGGAA	ATGTTGATCGTGGTGAGGTACTTGAG	
CL2617.Contig3	CAATGCCTACGAGTCAATCCCAATG	GCCAATGTAAGGTGACGCAGAAC	
CL3286.Contig1	GCACAATTAGTGGAACTTCACAACTCA	CCATCAGCCGCAAATCCTTCATAGA	
CL5004.Contig1	TCTACACGCACATACTACAGAGGAAC	TGGTGGTCTTCTTCTTGGTAACATCAT	
Unigene27389	GGTTGGTGGTGCCATGTCAGAAT	TCCCAGTTGCCATCTCTATGATTGTG	
CL2431.Contig1	GCAATCTTCACTTCGCCATTCTCAA	AATCGGATGCCAGACGCTACAC	
CL2431.Contig2	AGAACGAGATGGACTGCTGATATGC	CTTGGTGATGATGACGGTGGAGAT	
CL2431.Contig3	GAGATGGACGGCTGATATGTTATTGGA	CTTGGTGATGATGATGGAGATGATTGC	
CL2958.Contig3	GTGTTGGATGTACTGTGATTGAGATGG	AGGAATGGGAGGATGAGATTTAGTTGT	
CL10672.Contig3	GCCTAGACAGTTACCAAGAAGAAGACT	GCGTTTATGAATGCTACTCCCAACT	
CL7383.Contig1	TCAGCCAAGGAGAGGATCAGACG	CGACAATTGCTCACCACCACCATA	
Unigene35052	GCGATGGACGGCTAAAGAATTACTC	CCAGTACACAACTTGGAGAAGTCATAG	
CL3831.Contig3	TTTCTCCGAAGTAATAATGCCCGAAGA	GGGATGACAAGTATGGGTGACAAAGT	
Unigene25401	TCCTTGCATTCTCCTCAATAGTCATCT	GGTCATTGGGAAGGGCAGTAGTG	
Unigene21469	GCTCCAGTTCAAGTTCAACAACAGAG	GGAGACCTCGAACAAGTTACCACAC	
Unigene5108	TCAACTCTACCTTCATCGACAGTGTTC	CGTTCTTGCTCTCCATCTTTCCAGA	
Unigene9698	ACTCCGAACTCCGAGCACTGA	TCTCCGAATCATCATCTCCTTCTCATC	
CL8639.Contig2	TCACTCTCAACAAAGCCATCCAAGG	GTCCTCTGCCTGATCGTAATGAATGT	
Hb18S	GCTCGAAGACGATCAGATACC	TTCAGCCTTGCGACCATAC	Validation of reference genes for qRT-PCR in Coronatine-Induced Secondary Laticifer Differentiation
HbActin	GATTCCGTTGCCCAGAAGTC	CACCACTCAGCACAATGTTACC	
HbADF	GGAGAAGGCAAAGCAAGTC	GCATATCGGCACTCATCAG	
HbADF4	GTGCCGATATGCTGTCTATGATTT	TCCCTCTTGAACCTGTCCTTG	
HbCYP2	CTGTACTGCCAAGACTGAG	CTATGGCCTTCACCACATC	
beIF1Aa	GCGTGACTATCAGGACGACAA	CAAGACCTCCAGCAATACCCT	
HbeIF1Ab	TGGTGTTGGTGTGGTGATAG	TATTCCTGCCCATCTTCCTTG	

HbeIF2	CGACCTTTGATCCGTTTGCT	CTTCCTACCATTCCGTTGCT
HbeIF3	CGAAACCCAGATTCCTTCTACCT	CTGCACAGTAGTCAAGCTCTTTC
HbFP	ATCAGCTTGACGCAGGTTAC	GAAACAGACGCTTCTGAGTG
HbPTP	TGCCCTCCAATTCAACTG	TGCACCACAACGACATTC
HbRH2a	CCATCACCCAGGCTGTTATTTTC	GTAAAGTTATTGCTCCGCATCTTC
HbRH2b	AGGTGGATTGGCTAACTGAG	GAGCCCAAACATCAGTAGTG
HbRH8	TCACAGGGTTGGTAGATCAG	CCAAGCTCTTGCTCAATCC
HbROC3	ATGGAGCTATTCGCGGATAC	GGTGGAAACTCGATCCTTTG
HbTCPB	CAGACAGTCAATGACAGTAGGGTT	ATCCCTGCAGTGCAATCCTCC
HbUBC1	ACTTTGCCCTTGATGCCT	GCGTTAGTTGGGATCTGGT
HbUBC2a	CATTTATGCGGATGGAAGCA	CAGGGGAGTTTGGATTTGGA
HbUBC2b	CGACCAAGTTTTCATTTCGGGTG	AGTCTCTTCTTTGCTGGGGTTG
HbUBC3	CCCTGATGATCCACTTTCTG	ACCATCATGCACCACTTG
HbUBC4	TCCTTATGAGGGCGGAGTC	CAAGAACCGCACTTGAGGAG
HbYLS8	CCTCGTCGTCATCCGATTC	CAGGCACCTCAGTGATGTC
